# Modulation of alternative splicing during early infection of human primary B lymphocytes with Epstein-Barr virus (EBV): a novel function for the viral EBNA-LP protein

**DOI:** 10.1093/nar/gkab787

**Published:** 2021-09-16

**Authors:** Evelyne Manet, Hélène Polvèche, Fabrice Mure, Paulina Mrozek-Gorska, Florian Roisné-Hamelin, Wolfgang Hammerschmidt, Didier Auboeuf, Henri Gruffat

**Affiliations:** CIRI, Centre International de Recherche en Infectiologie, RNA Expression in Viruses and Eukaryotes Group, Univ Lyon, Université Claude Bernard Lyon I, INSERM U1111, CNRS UMR5308, ENS Lyon, Lyon F-69007, France; CECS, I-Stem, Corbeil-Essonnes F-91100, France; CIRI, Centre International de Recherche en Infectiologie, RNA Expression in Viruses and Eukaryotes Group, Univ Lyon, Université Claude Bernard Lyon I, INSERM U1111, CNRS UMR5308, ENS Lyon, Lyon F-69007, France; Research Unit Gene Vectors, Helmholtz Zentrum München, German Research Center for Environmental Health and German Center for Infection Research, D-81377 Munich, Germany; CIRI, Centre International de Recherche en Infectiologie, RNA Expression in Viruses and Eukaryotes Group, Univ Lyon, Université Claude Bernard Lyon I, INSERM U1111, CNRS UMR5308, ENS Lyon, Lyon F-69007, France; Research Unit Gene Vectors, Helmholtz Zentrum München, German Research Center for Environmental Health and German Center for Infection Research, D-81377 Munich, Germany; CNRS UMR 5239, ENS de Lyon, Lyon F-69007, France; CIRI, Centre International de Recherche en Infectiologie, RNA Expression in Viruses and Eukaryotes Group, Univ Lyon, Université Claude Bernard Lyon I, INSERM U1111, CNRS UMR5308, ENS Lyon, Lyon F-69007, France

## Abstract

Epstein-Barr virus (EBV) is a human herpesvirus associated with human cancers worldwide. *Ex vivo*, the virus efficiently infects resting human B lymphocytes and induces their continuous proliferation. This process is accompanied by a global reprogramming of cellular gene transcription. However, very little is known on the impact of EBV infection on the regulation of alternative splicing, a pivotal mechanism that plays an essential role in cell fate determination and is often deregulated in cancer. In this study, we have developed a systematic time-resolved analysis of cellular mRNA splice variant expression during EBV infection of resting B lymphocytes. Our results reveal that major modifications of alternative splice variant expression appear as early as day 1 post-infection and suggest that splicing regulation provides—besides transcription—an additional mechanism of gene expression regulation at the onset of B cell activation and proliferation. We also report a role for the viral proteins, EBNA2 and EBNA-LP, in the modulation of specific alternative splicing events and reveal a previously unknown function for EBNA-LP—together with the RBM4 splicing factor—in the alternative splicing regulation of two important modulators of cell proliferation and apoptosis respectively, *NUMB* and *BCL-X*.

## INTRODUCTION

Epstein-Barr virus (EBV) is a ubiquitous herpesvirus associated with several human cancers including Burkitt's lymphoma, Hodgkin's disease (HD), rare T-cell and NK-cell lymphomas, undifferentiated nasopharyngeal carcinoma (NPC), and other lymphomas such as post-transplant lymphoproliferative disease in the immuno-compromised (for a review see (1)). Primary infection is usually asymptomatic in childhood but can result in Infectious Mononucleosis (IM) later in life. Upon infection, the virus persists in a life-long, latent state in the memory B cells of infected individuals, with intermittent viral production in the oropharynx (2). *Ex vivo*, EBV has the unique capacity to induce growth transformation of resting primary human B-lymphocytes upon their infection leading to the establishment of lymphoblastoid cell lines (LCLs). In such cell lines, most of the cells do not produce virions but express nine viral proteins—six nuclear antigens (EBNA 1, 2, 3A, 3B, 3C and LP) and three membrane proteins (LMP 1, 2A and 2B)—as well as non-coding RNAs such as EBER1, EBER2, a snoRNA and 44 miRNAs. These viral factors act in concert to ensure robust cell proliferation and survival (3).

Detailed study of the early events following EBV infection of purified human B lymphocytes has revealed that virally infected B-lymphocytes first become activated and then grow massively in size but do not start to proliferate immediately. This primary step lasts until about 72 h post infection (p.i.), when the activated cells start to synthesize cellular DNA before they initiate the first round of cell division (96 h p.i.) with remarkable synchronicity. A short phase of hyperproliferation ensues, during which the cells divide with a short generation time of around 12 h. Hyperproliferation peaks at day 5 p.i. Thereafter—7 to 8 days p.i.—cell division slows down, reaching a generation time of about 30 h (4). At the molecular level, it has been shown that primary resting B cells transiently express many viral genes upon initial infection—including some genes typically expressed during EBV’s lytic phase. Viral expression of these lytic genes can be due to the transfer of viral mRNAs, which are contained as cargo in the virus particle and which are immediately translated upon infection (5). Known cargo mRNAs include viral transcripts that contribute to early immune evasion and cellular activation. Moreover, upon infection, the viral DNA is epigenetically naïve—i.e. free of methylated cytosines—and lacks nucleosomal structures and associated histones. As a consequence, many viral genes are transiently transcribed upon nuclear delivery of the viral genomic DNA until repressive chromatin is established to restrict viral gene expression to latency-associated genes only (6). The first genes to be immediately expressed after B cell infection are EBNA2 and EBNA-LP. Together, they have been shown to play a crucial role during the early phase of infection. EBNA2 is essential to induce cellular proliferation of newly infected B-lymphocytes whereas EBNA-LP contributes to cell activation and proliferation of the resting cells (7–10). Finally, EBNA2 and EBNA-LP proteins have previously been shown to cooperate and promote G_0_ to G_1_ transition of resting B-lymphocytes upon initial infection with EBV (11).

In the early phase of infection, EBV drives both cell activation and cell division in a two-step process. These events imply a complete reprogramming of gene expression in the resting, quiescent B cells. Up to now, most studies have focused on the impact of EBV infection on gene transcription (4,12,13), but mRNA levels do not only depend on transcription but also on co- and post-transcriptional events that can generate diverse splice variants. Recent genome-wide studies have demonstrated that as much as 94% of all human protein-coding genes generate multiple mRNA splice variants. Alternative splicing provides a means to regulate the localization, stability and translation of mRNAs and greatly increases the diversity of proteins encoded by a limited number of genes within cells (14,15). In addition to increasing the diversity of proteins, alternative splicing can result in the production of mRNAs that are rapidly degraded by the Nonsense-Mediated mRNA Decay (NMD) pathway, thereby contributing to the regulation of gene expression levels (16). Different cell types are thus characterized by particular splicing programs, together with specific transcription programs (17). Coordinated regulation of alternative splicing plays a key role during development as well as in cellular responses triggered by the environment (e.g. stress; (18)). Recent genome-wide analyses also suggest that modifications of splicing programs play a crucial role in cancer development (19–22) and the dynamics of RNA splicing is the basis for bioinformatic evaluation of cell fate at the level of single cells (23). Moreover, several human genetic diseases are thought to be caused by aberrant mRNA splicing further illustrating the importance of correct splicing regulation (24). Except for a paper by Homa *et al.* (25) that compared primary B cells from several donors with their EBV-infected derived LCL counterparts, little is known about the impact of EBV infection on alternative splicing.

In the present study, we have identified important, previously unknown changes in splice variant expression during the early phase of B-cell infection with EBV. Analysis of our data suggests that modifications in the alternative splice mRNA variant expression pattern occur as early as day 1 post-infection (p.i.). At this very early stage, a large fraction of changes are predicted to either modify the capacity of the resulting mRNA isoform to be recognized as a target for NMD or to produce truncated proteins. Our results thus strongly suggest that splicing regulation potentially provides an additional mechanism of regulating gene expression upon EBV-induced B cell activation and proliferation. By using EBV derivatives incapable of expressing EBNA2 or EBNA-LP—the first two latency-associated viral genes to be expressed upon infection—we have demonstrated that certain changes in alternative splice variant expression are directly or indirectly regulated by either EBNA2 or EBNA-LP. Finally, by using reporter assays, our results reveal a previously unknown function of EBNA-LP in modulating alternative splicing.

## MATERIALS AND METHODS

### Cell culture and transfection

HEK293T cells were grown at 37°C in DMEM supplemented with 10% foetal calf serum (FCS). DG75, primary and immortalized B cells were cultured in RPMI 1640 medium (Gibco–Invitrogen Life Technologies) supplemented with 10% FCS. Plasmid transfection of HEK293T cells was performed using either PEI (Polysciences) or jetPEI^®^ (Polyplus-Transfection) transfection reagents. Cells were collected 48 h post-transfection except for the GST-pulldown experiments of endogenous RBM4 and RBM10 for which cells were collected 24 h post-transfection. Plasmid transfection of DG75 cells was performed by using the Neon™ Transfection System (Invitrogen) (microporation parameters: Tip 10 μl, 1350 V, 30 ms, 1 pulse).

### Virus production, cell infection and sample preparation for RNA sequencing

Producer cell clones HEK293 carrying wt/B95.8 EBV (2089), EBNA2 KO (3057) and EBNA-LP KO (8060) recombinant viruses (10) were transfected with a BZLF1 expression plasmid to induce the viral productive cycle and a BALF4 expression vector to increase the virus titer, as previously described (26). Virus supernatants were harvested three days post-transfection, filtered through a 0.45 μm filter and stored at 4°C. Viral titers were determined by infecting 2.5 × 10^5^ Raji cells with increasing dilutions of virus supernatants. Three days after infection, GFP-positive Raji cells were visualized using a fluorescent microscope and counted by FACS analysis. B lymphocyte purification, infection, sample collection and library preparation for RNA sequencing has been described in detail in Mrozek-Gorska *et al.* (4). Briefly, human B-lymphocytes were prepared from adenoid tissues from three donors, then sorted by FACS to enrich for naïve B-cells prior to infection with wt EBV (2089) with a multiplicity of infection (MOI) of 0.1. 1 × 10^6^ cells were collected at day 0 prior to infection and at days 1, 2, 3, 4, 5, 8 and 14 p.i. after FACS sorting of living cells. Cells were then washed twice in PBS, resuspended in Trizol reagent and snap frozen in liquid nitrogen. Total RNA was extracted from 1 × 10^6^ frozen Trizol samples and further purified using the RNAeasy kit (Qiagen) including DNAse treatment. The oligodT primed cDNA libraries were generated at Vertis Biotechnology AG. All prepared libraries were sequenced together (paired-end, 100 bases) using an Illumina HiSeq4000 platform (Institute of Human Genetics, Helmholtz Zentrum München) (4). For RNA-seq validations, human peripheral B cells purified from blood samples using the RosetteSep human enrichment kit (Stemcell Technologies; 15064) were infected with wt EBV or EBNA2 or EBNA-LP KO mutant EBVs and collected at different days p.i. Viable, infected B cells were physically sorted by FACS, using size and granularity criteria before RNA extraction. The newly established Lymphoblastoid Cell Line (LCL) used in the validation experiments was collected 3–4 weeks after infection and the cells were diluted with fresh medium to 7 × 10^5^ cells/ml on average 24 h before harvest.

### Bioinformatic analysis

**Figure 1.**
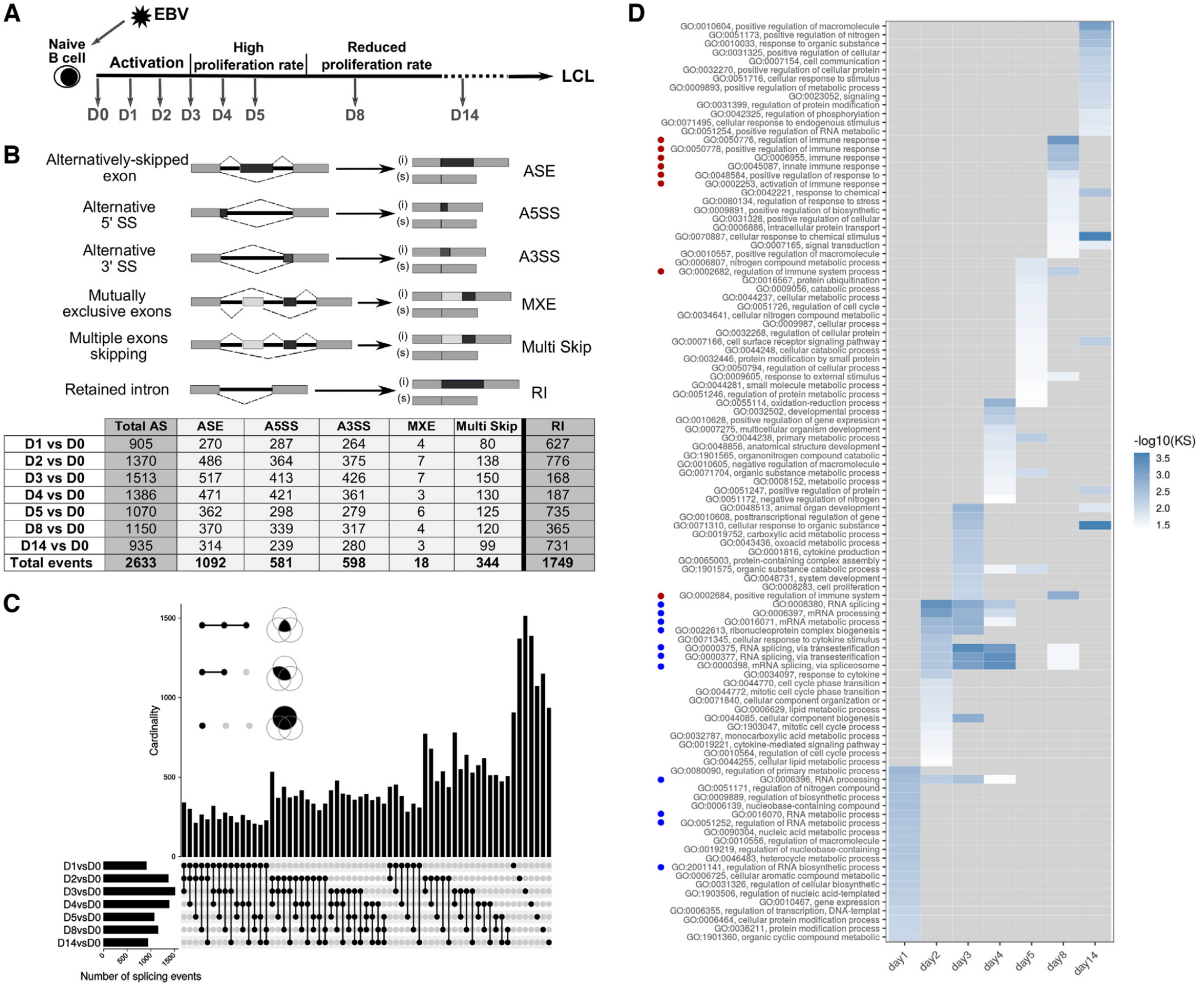
Changes in alternative splice variant expression in B cells infected with EBV at different time points post-infection. (**A**) Schematic representation of the kinetics of infection of B-lymphocytes with EBV and experimental scheme of RNA-seq. Following EBV infection, naïve B-lymphocytes are immediately activated and their cell volumes increase. The first cell division occurs 4 days post-infection (p.i.). A short phase of hyperproliferation ensues until day 6, during which cells divide with a generation time of 8–12 h. Thereafter, cell division slows down, reaching a generation time of about 30 h, 7–8 days p.i., ultimately leading to the establishment of lymphoblastoid cell lines (LCLs) (92). For RNA-seq experiments, quiescent naïve B-lymphocytes (IgD+, CD38–) purified from human adenoids were infected with EBV as described in (4). Cells were collected prior to EBV infection (D0) then every day p.i., during the activation phase (D1, D2, D3), at the beginning of proliferation (D4 and D5) and finally at days 8 and 14 p.i. (D8 and D14). **(B)** Quantification of the different splicing events at the indicated time points compared with non-infected B cells. The six different categories of splicing events studied are schematically represented. (i) indicates inclusion, (s) indicates skipping. The number of differential splicing events identified at each time point versus non-infected cells (D0) is summarized in the table below. Total AS corresponds to the total number of differential ASE, A5SS, A3SS, MXE and Multi Skip events. The number of differential retained intron events are summarised in a separate column on the right of the table. The bottom row of the table called ‘Total events’ summarizes the total number of independent events for each alternative splicing category. (**C**) The UpSet Plot visualizes the intersecting sets of AS events at different time points after EBV infection. As insets, the three Venn diagrams illustrate the syntax of the UpSet diagram. The single dots and dots with interconnecting vertical lines form the intersection matrix. Paired intersections are depicted as black dots; gray dots indicate the sets that are not part of the intersection. Black lines connecting 2 or more black dots indicate which sets form the intersections. In the lower part, groups of AS events, obtained from the pairwise comparison as indicated (D1vsD0, etc.) form seven sets in the UpSet matrix, which are depicted as black horizontal bars with ‘Number of splicing events’ to indicate the set sizes. Each row represents a matrix set. The columns show the intersections depicted as areas in the schematic Venn diagram as explained in the inset of this panel. The height of the black columns indicates the cardinality (number of elements) in the different intersections. (**D**) Gene ontology (GO) enrichment analysis performed on human genes for which differential ASE events were identified upon EBV-infection. Only GO terms corresponding to at least 20 genes were considered. –log_10_ of *P*-values, calculated using the Kolmogorov–Smirnov (KS) test, are represented by a colour code as indicated on the right of the table. Grey boxes correspond to a lack of enrichment of the GO term at the indicated day post-infection. GO terms related to RNA processing or the immune response are indicated by blue and red dots, respectively.

RNA-seq data from three biological replicates for each condition were analysed using FaRLine (FasterDB RNAseq Pipeline), to identify alternatively skipped exons (ASE), alternative 3′ splice sites (A3SS), alternative 5′ splice sites (A5SS), mutually exclusive exons (ME) and multiple exons skipping (Multi Skip) (Figure 1B) as described in Benoit-Pilven *et al.* (27). Briefly, reads crossing the exon–exon junctions (‘junction reads’) were extracted from the read alignment files to analyse splicing events annotated in FasterDB, a database containing all annotated human splicing variants (28). The differential analysis works on pairs of splicing variants (e.g. skipped versus included exons) for which read counts are available in each replicate of each condition, and tests whether a variant is enriched relative to the other in a particular condition. Counts are modeled using a negative binomial distribution. FaRLine fits a generalized linear model and tests for the effect of an interaction between the variant and the condition, using a likelihood ratio test with a 5% false discovery rate (FDR), to control for multiple testing. A percent splicing index (PSI) value is calculated for each sample as the ratio of inclusion junction reads to the sum of inclusion and exclusion junction reads. As the datasets are paired, the difference in PSI values for each event (ΔPSI) is calculated as the median of ΔPSI values for each replicate. A filter is then applied on exon skipping events detected to select significant variants with an adjusted *P* valuec ≤0.05 and a ΔPSI value ≥10%. The intersections of splicing events between any given pair of time points was visualized by an UpSet plot (UpSetR R package, version 1.3.3) (29). Retained introns (RI) regulated between conditions were determined using the Multivariate Analysis of Transcript Splicing (rMATS) program (30) with the same ΔPSI cutoff value as above. Enrichment analysis for Gene Ontology (GO) was performed using the ’topGO’ R package (Alexa and Rahnenfuhrer, 2019. topGO: Enrichment Analysis for Gene Ontology; R package version 2.38.1.). The ‘classic’ algorithm was used and *P* values calculated using the ‘Kolmogorov–Smirnov’ test. Only GO terms corresponding to at least 20 genes in the package reference ‘org.Hs.eg.db’ were retained (Marc Carlson, 2019, org.Hs.eg.db: Genome-wide annotation for Human; version 3.10.0). Graphics were realized using the R ‘ggplot2’ package (H. Wickham. 2016. ggplot2: Elegant Graphics for Data Analysis; Springer-Verlag New York)

### Plasmids

The pSG5-EBNA2 expression vector has been described elsewhere (31). Expression vectors for EBNA-LP, RBM4, RBM5, RBM6 and RBM10, tagged with the Flag or the myc epitope were generated using the Gateway Recombinational Cloning system (Thermo Fischer Scientific). For this, the respective coding sequences were PCR-amplified (KOD Hot Start DNA Polymerase^®^, EMD Millipore) either from cDNA generated by reverse transcription, using the qScript™ cDNA synthesis kit (Quanta Biosciences; for EBNA-LP, RBM4 and RBM5) or from the pDEST26-RBM6 and pDEST26-RBM10 plasmids (for RBM6 and RBM10; kind gift from Dr Valcarcel (32). Oligonucleotides used for these amplifications are listed in Supplementary Table S9. The PCR-amplified DNA fragments were first cloned into pDONR207, then transferred into pCI-neo-3xFlag-gw, pDEST-myc (33) or pDEST^TM^27 (Invitrogen). It should be noted that the EBNA-LP construct contains 3 W_1_-W_2_ repeats. The pCMV-RG6-Numb plasmid was a kind gift from Dr Valcarcel (32). pCMV-RG6-Numb is a modified version of the splicing reporter RG6 minigene (34) generated by replacing a model cassette exon by the human *NUMB* exon 11 together with 100 and 50 nucleotides of its flanking intronic sequences respectively (Figure 4A), thereby minimizing the alternatively spliced region of *NUMB* and its potential key regulatory elements. For generating pLMP-RG6-Numb, the RG6-Numb complete reporter gene was amplified from pCMV-RG6-Numb using the primers listed in Supplementary Table S9, and cloned by In-Fusion (In-Fusion® PCR cloning kit, Clontech) between the pLMP-Luc (35) Xho I and Xba I restriction sites, in place of the luciferase gene. The *BCL-X* minigene (*BCL-X* miniG) construct was a kind gift from Dr Chalfant (36). The mutated *BCL-X* R4m miniG, was generated by site-directed mutagenesis (QuickChange Site-Directed Mutagenesis kit, Agilent) using the primers listed in Supplementary Table S9. pLKO.1-shRBM4 plasmid (mission shRNA TRCN0000164640) was supplied by Sigma-Aldrich.

### RNA analysis

RNAs were prepared using the Nucleospin RNA/protein kit (Macherey-Nagel) and reverse transcribed using the qScript™ cDNA synthesis kit (Quanta Biosciences). Standard PCRs were performed using the GoTaq®DNA polymerase (Promega) and the PCR-amplified fragments were analysed on 2.5% agarose gels. Gels were analysed on a Gel Doc™ XR + Imaging System (Bio-Rad) and quantification performed using Image Lab software (Bio-Rad). Quantitative PCR (qPCR) was performed using FastStart Universal SYBR Green Master (Rox); Roche Molecular Biochemicals) on an Applied Biosystems 7000 thermocycler. Cycling conditions were 5 min at 95°C, 45 cycles of 15 s at 95°C and 30 s at 60°C in a 96-well thermoblock. This program was followed by a melting curve analysis in order to verify the specificity of the PCR products. For RNA-seq validations, we selected 88 ASE events based on the alternative event occurring at days 1 or 2 p.i., their ΔPSI being in the top range and the length of their alternative exon being compatible with efficient standard PCR amplification of both isoforms using primers located within the adjacent constant exons. The sequence of the primer pairs and the expected size of each isoform are listed in Supplementary Table S9. Among the 88 selected ASE events, 28 could not be validated for technical reasons, either because the specific primer pairs did not yield any amplification products, or because the difference in quantity between the inclusive and skipping isoforms was too high to allow the visualization of both isoforms from the same PCR reaction. In four cases, primer pairs did not amplify products of the expected size. For analysis of the reporter minigene transcripts, primers used to selectively amplify different isoforms of the RNAs were generated such that one of the primer overlapped the exon junction but with only a 5 nt overlap with one of the exons. The various sets of primers used in the study are listed in Supplementary Table S9.

### GST pull-down assays

HEK293T cells were transfected with the Glutathione-S-transferase (GST) fusion proteins and Flag-tagged proteins. 1–5 × 10^6^ cells were collected either 48 h post-transfection for the overexpressed RBM proteins pulldown experiments—or 24 h post-transfection for the endogenous RBM proteins pulldown experiments—then lysed for 30 min at 4°C in 500 μl GST-pull down buffer (20 mM Tris pH 8, 150 mM NaCl, 1mM EDTA, 0.5% NP40) plus protease inhibitors (Roche Molecular Biochemicals). The lysates were subsequently sonicated to reduce viscosity and clarified by centrifugation. 40 μl of a 50:50 Glutathione-Sepharose 4B beads (GE Healthcare) suspension, preincubated with 1 mg/ml bovine serum albumin (BSA) for 2 h and resuspended in GST-pulldown buffer, was added to the lysates in order to purify the GST-fusion proteins and their associated proteins. After 4 h incubation at 4°C, the beads were washed 5-fold in GST-pull down buffer. RNAse A (10 mg/ml) was added to the fourth wash for a 15 min incubation period at RT. Bound proteins were fractionated by SDS-PAGE and analysed by Western blotting.

### Western blot analysis

Proteins from transfected cells were obtained using the Macherey Nagel RNA/protein kit, from the same cell extracts that yielded the RNA preparation, and analysed by western blotting using Hybond ECL membranes (GE Healthcare Life Science). Blots were incubated either with anti-Flag M2 (Sigma-Aldrich), anti-EBNA2 (PE2 Abcam), anti-GST-Tag (GST.B6/G2R, Covalab), anti-RBM4 (Proteintech), anti-αTubulin (Sigma-Aldrich) monoclonal antibodies or anti-RBM10 (Sigma-Aldrich) polyclonal antibody. Anti-rabbit and anti-mouse (HRP)-conjugated antibodies (GE Healthcare Life Science) were used as secondary antibodies. Blots were revealed with either Clarity™ Western ECL Substrate (BioRad) or the ECL Select™ Westen Blotting Detection Reagent (Amersham) and analysed using a ChemiDoc™ Imaging System (Bio-Rad). Quantification was made using Image Lab software (Bio-Rad).

### Proximity ligation assay (PLA)

PLA detection was carried out using the Duolink In Situ Red Fluorescence kit (Sigma-Aldrich) according to the manufacturer's instructions. Briefly, PFA-fixated HEK293T cells expressing Flag-EBNA-LP and myc-tagged-RBM4 were blocked for 1 h at room temperature. Primary antibodies were added at a dilution of 1:200 (mouse anti-myc 9E10, Covance, Inc.) and 1:500 (rabbit anti-Flag, Sigma-Aldrich) in 40 μl Duolink antibody diluent and incubated at 4°C overnight. Samples were washed twice with Wash Buffer A for 5 min each, then secondary antibodies (Duolink anti-mouse PLA-minus probe and Duolink anti-rabbit PLA-plus probe) were added for 1 h at 37°C. After two washes with Wash Buffer A, ligation mix was added, for 30 min at 37°C and the samples washed again twice with Wash Buffer A. The amplification reaction was carried out at 37°C for 100 min. Slides were subsequently washed twice with 1 x Wash Buffer B, once with 0.01× Wash Buffer B and mounted with Duolink In Situ Mounting Medium with DAPI. Microscopic examination was performed using an AxioImager Z1 epifluorescence microscope (Zeiss). Images were analysed using FIJI/ImageJ software.

## RESULTS

### Overall regulation of cellular alternative splicing upon infection of primary B lymphocytes with EBV

**Figure 2.**
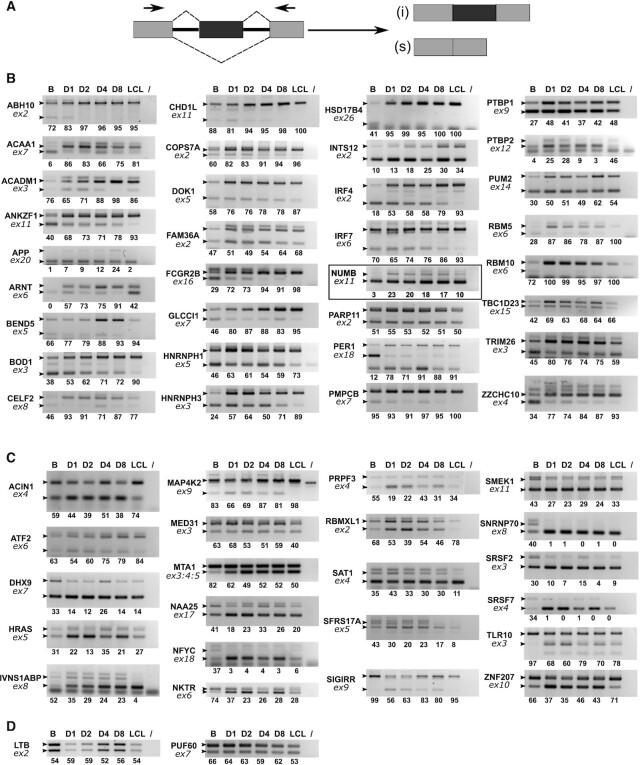
RT-PCR validation of ASE events. (**A**) schematic representation of a typical ASE and primer positions for PCR amplification. (**B** and **C**) validation of ΔPSI positive and negative events respectively. Human primary B cells were infected with EBV and cells collected prior to infection (D0), at days 1, 2, 4 or 8 p.i. (D1, D2, D4 and D8 respectively) and after establishment of the LCL. RNA was analysed by semi-quantitative RT-PCR followed by agarose gel migration of the amplification products. Gene names are indicated on the left of each panel, together with the number of the concerned exon (ex: exon). Small black arrow heads indicate the expected migration location of each isoform as determined using the FasterDB database. The last lane of each panel (/) corresponds to a PCR amplification control performed in the absence of cDNA. The relative amounts indicated under each panel represent the percentage of inclusive isoform and were quantified using Image Lab software (Bio-Rad). The highlighted panel signals a splicing event that will be further studied as a putative target for EBNA2 and EBNA-LP regulation. (**D**) Examples of non-validated events.

In order to investigate the impact of EBV on cellular mRNA splicing and its regulation during infection of primary B-lymphocytes, we performed a time course experiment monitoring cellular mRNA splice variants at different times post-infection (p.i.). For this, we conducted high-throughput paired-end RNA sequencing (RNA-seq) with RNA from quiescent naïve human B cells (IgD+/CD38- fraction) that had either been mock-infected or infected with the wt/B95.8 (2089) EBV strain and harvested 1, 2, 3, 4, 5, 8 or 14 days p.i. (Figure 1A). RNA-seq data generated from three biological replicas were previously analysed to monitor global transcriptional changes (4) (https://scialdonelab.shinyapps.io/EBV_B/). Using the same data set, we studied the differential alternative splicing events including alternatively-skipped exon or cassette exon (ASE), alternative 5′ or 3′ splice sites (A5SS or A3SS), mutually exclusive exons (MXE), multiple-exons skipping (Multi Skip) as well as retained intron (RI) events occuring in the course of infection by using the FaRline pipeline (27) and rMATS program (30) (Figure 1B). A total of 4382 differential independent splicing events—2633 corresponding to alternative splicing, 1749 to intron retention—was identified with significant difference in Percent-Spliced-In (PSI) values (ΔPSI ≥ 0.1) in the course of EBV infection (Figure 1B and Supplementary Tables S1 to 8). Altogether, 2928 genes underwent changes in alternative mRNA splice variant expression. Significant and consistent changes were detected as early as day 1 p.i. Most changes appeared during the first days of infection and many were conserved thereafter (UpSet plot, Figure 1C and Figure 2). When analysing the cellular function of genes affected by changes in alternative splice variant expression, we found a significative enrichment in GO functions associated with mRNA processing and its regulation at the very beginning of infection at day 1, which increased during day 2–4 p.i. (Figure 1D). Later and primarily at day 8 p.i., a significative enrichment in GO functions associated with the immune response and its regulation appears to be predominant (Figure 1D).

Focusing on alternative events that occurred during the first few days after infection, we selected 88 ASE events to be validated by semi-quantitative PCR after reverse transcription (RT-PCR) as indicated in Figure 2A. Changes in alternative mRNA splice variant expression were compared between non-infected primary B cells, B cells infected for 1, 2, 4 or 8 days p.i. and a newly established cell line (LCL) derived from the same donor. Out of 60 ASE candidate events which we could analyse, 56 were confirmed, resulting in a validation rate of 93%. Figure 2B gathers ΔPSI positive events (i.e. corresponding to an increase in alternative exon inclusion following EBV infection) while Figure 2C gathers ΔPSI negative events (i.e. corresponding to a decrease in alternative exon inclusion). As expected from the GO analysis (Figure 1D) many analysed genes such as *DHX9*, *RBM5*, *RBM10*, *SRSF2*, *HNRNPH1*, *RBMXL1*, *SNRNP70*, *PRPF3*, *HNRNPH3*, *PTBP1* and *CELF2* have functions involved in mRNA processing. As was the case for our RNA-seq results as a whole, nearly 60% of the validated ASE events showed an increase in exon inclusion following EBV infection (ΔPSI positive events in Figure 2B). When we analysed the lengths of the exons involved in the 56 validated ASE events, it appeared that 37 were non-symmetrical exons (i.e. their lengths are not a multiple of three nucleotide residues). Therefore, in the case of coding exons—i.e. exons that are part of the translated regions of mRNAs—skipping or inclusion will introduce frameshifts in their coding sequences potentially leading to (i) translation of a completely different protein or a protein that differs in part of its sequence; (ii) translation of a truncated protein; or (iii) encounter of a Premature Stop Codon (PTC) that will cause mRNA degradation by the Nonsense-Mediated mRNA Decay (NMD) pathway. We used the FasterDB database (http://fasterdb.lyon.unicancer.fr/; (28)) to predict the occurrence of a truncated protein or the presence of PTCs potentially leading to NMD, in one of the alternative isoforms of 19 of our validated ASE events. In addition, we also identified nine cases of alternative poison exon (i.e. symmetrical exon that carry in frame PTC in its sequence) (Table 1). Interestingly, 26 out of 28 of these ‘non-productive’ RNA isoforms are expressed in non-infected primary B cells suggesting that the corresponding genes are actively transcribed in these cells but probably without resulting in the expression of the related proteins. It is only upon EBV infection that changes in splicing regulation would lead to the production of the alternative ‘productive’ RNA isoforms without needing *de novo* transcriptional activation.

**Table 1.** Putative functional impact of alternative splicing of both non-symmetrical exons and poison exons in primary B or EBV-infected B cells. Non-symmetric exons as well as PTC-containing ASEs (poison exons) from our panel of validated events were identified using FasterDB (http://fasterdb.lyon.unicancer.fr/; (83)). Affected exons are identified by their given number in FasterDB, and the corresponding ΔPSI sign indicated. In several cases, the predicted impact (i.e. NMD trigger or truncated protein genesis) has previously been reported, thereby supporting our predictions. Corresponding bibliographic references are as follows: (a) Fidaleo *et al.* (84); (b) Hubert *et al.* (85); (c) Barbier *et al.* (86); (d) Boutz *et al.* (87); (e) Sun *et al.* (88); (f) Moutarda-Maarabouni *et al.* (89); (g) Hyvönen *et al.* (90); (h) Lareau *et al.* (66); (i) Sureau *et al.* (91); (j) Ni *et al.* (68). * indicates an ASE not reported in FasterDB.

Gene symbol	exon	ΔPSI	Primary B cells	Infected B cells
ACAA1	7	+	NMD	
BEND5*	5	+	NMD	
CHD1L*	11	+	NMD	
HNRNPH1	5	+	NMD ^(i)^	
HNRNPH3*	3	+	NMD	
HRAS	5	-	NMD ^(c)^	
HSD17B4*	26	+	NMD	
PMPCB*	7	+	NMD	
PTBP2	12	+	NMD ^(d, i)^	
RBM5	6	+	NMD ^(e)^	
RBM10	6	+	NMD ^(e)^	
SIGIRR*	9	-		NMD
SRSF2	3	+	NMD ^(h, i)^	
DHX9	7	-	NMD/poison exon ^(a)^	
IVNSIABP	8	-	NMD/poison exon	
NAA25	17	-	NMD/poison exon	
PARP11	2	+		NMD/poison exon
PRPF3	4	-	NMD/poison exon	
SAT1	4	-	NMD/poison exon ^(g)^	
SFRS17A	5	-	NMD/poison exon	
SNRNP70	8	-	NMD/poison exon	
SRSF7	4	-	NMD/poison exon ^(h, j)^	
ABHD10	2	+	N-ter truncated protein	
IRF4	2	+	N-ter truncated protein	
IRF7	6	+	N-ter truncated protein	
NKTR	6	-	N-ter truncated protein	
DOK1	5	+	C-ter truncated protein ^(b)^	
RBM5	6	+	C-ter truncated protein ^(f)^	
FAM36A	2	+	Changes the coding frame	
MED31	3	-	Changes the coding frame	

Taken together, these results indicate that substantial remodelling of alternative splicing occurs very early after infection of primary B cells with EBV. Interestingly, our results with a panel of validated genes suggest that, in primary B cells, actively transcribed genes code for alternatively spliced mRNAs that either do not lead to protein synthesis because they are targeted to NMD prior to their translation or lead to the potential production of truncated proteins. Therefore, EBV infection of primary B cells not only induces drastic changes in the B cell transcriptome as described by Mrozek-Gorska *et al.* (4) but also induces major changes in the alternative splicing pattern of cellular transcripts.

### Certain alternative splicing events appear to depend on the expression of the EBV proteins EBNA2 or EBNA-LP

**Figure 3.**
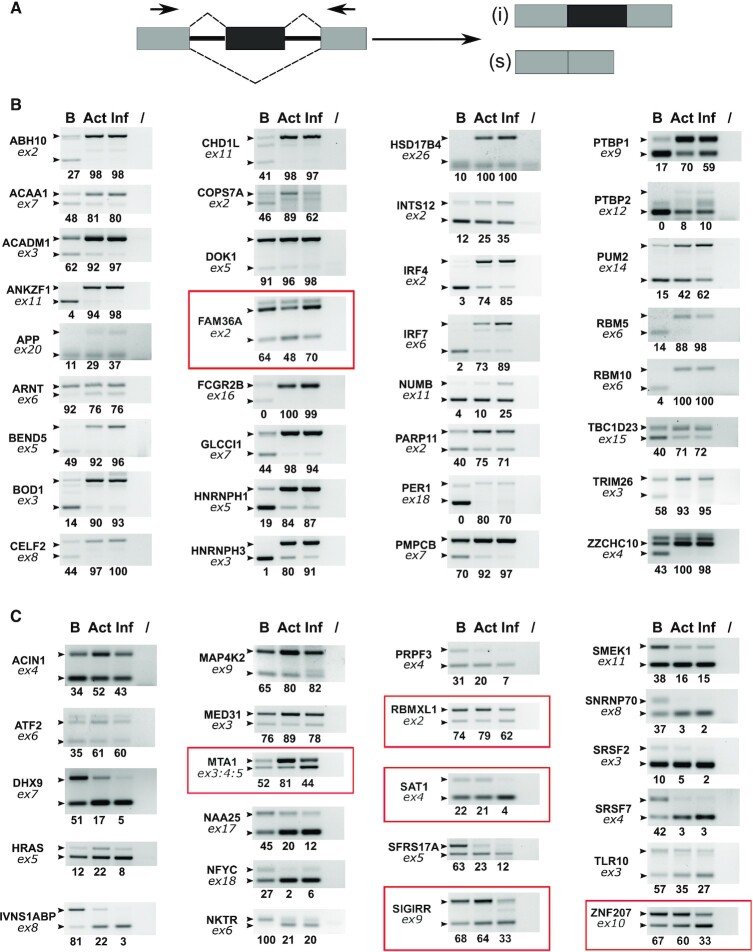
Comparative study of the impact of CD40L/IL4 B cell activation versus B cell infection by EBV on splicing regulation. (**A**) schematic representation of a typical ASE and primer positions for PCR amplification. (**B** and **C**) Primary B lymphocytes from the same donor were either non-treated (lane B) or treated with CD40L plus IL4 (lane Act) or infected by EBV for 72 h (lane Inf). RNA was analysed by semi-quantitative RT-PCR followed by agarose gel analysis of the amplification products. Gene names are indicated on the left of each panel, together with the number of the concerned exon (ex: exon). Small black arrow heads indicate the expected migration location of each isoform as determined using the FasterDB database. The final lane of each panel (/) corresponds to a PCR amplification control performed in the absence of cDNA. The relative amounts indicated under each panel represent the percentage of inclusive isoform and were quantified using Image Lab software (Bio-Rad). The panels highligted in red indicate splicing event changes that differ between CD40L/IL4 treated cells and EBV infected B cells.

We have seen that EBV infection drastically affects cellular gene expression in human B-lymphocytes both at the level of global transcription (4) and at the level of alternative splice mRNA variant expression (this article) within the first few days of infection. Using our panel of validated splicing events, we then asked whether some of the splicing changes induced by EBV infection were also induced by the activation of primary B cells with a combination of CD40 ligand (CD40L) and Interleukin 4 (IL4) (37). As shown in Figure 3, at 72 h p.i. or post treatment, a majority of the splicing changes induced by the EBV infection were also induced by the CD40L/IL4 treatment of the cells. Interestingly, however, some of the splicing changes, such as those of FAM36A, MTA1, RBMXL1, SAT1, SIGIRR and ZNF207 appeared to be specific of EBV infection. As EBNA2 and EBNA-LP are the first latency-associated EBV genes that are transcribed and expressed following B-cell infection (11) we next evaluated whether some of the observed alternative splicing events were dependent on the expression of EBNA2 or EBNA-LP or both. For this, human primary B-lymphocytes were infected with wt EBV or mutant EBVs devoid of EBNA2 or EBNA-LP (10). As previously reported, cells infected with the EBNA2 KO EBV appeared to be partially activated, however, they did not enter the cell cycle and began to die 96 h p.i. By contrast, cells infected with the EBNA-LP KO mutant EBV were activated and started to enter the cell cycle even though cell proliferation was substantially reduced and inefficient as reported previously (9,10). Cells infected with these two EBV mutants were collected 72 h p.i. and RNA was analysed by semi-quantitative RT-PCR to evaluate changes in the ASE ratio of our validated transcript panel.

**Figure 4.**
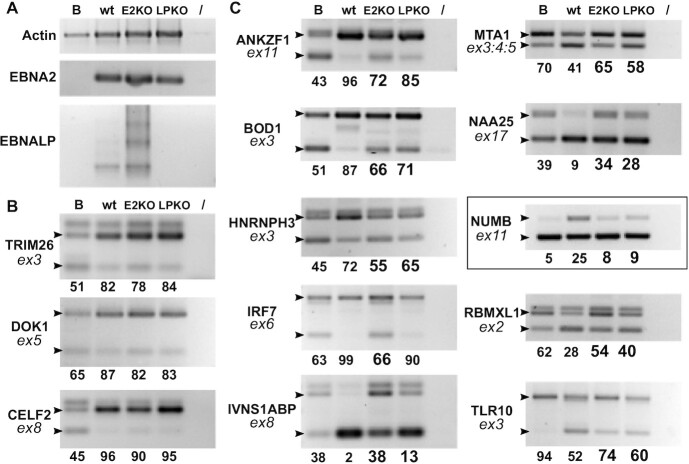
Impact of EBNA2 and EBNA-LP KO on the occurrence of specific validated ASE events upon primary B cell infection. Primary human B cells from the same donor were concomitantly infected with either wt, EBNA2-KO or EBNA-LP-KO EBV. Part of the cells were collected prior to infection and then at 72 h p.i. RNA was analysed by semi-quantitative RT-PCR. (**A**) RT-PCR using primers specific for actin and the viral EBNA2 and EBNA-LP RNAs. (**B**) Examples of ASE events that are not affected by the absence of either EBNA2 or EBNA-LP. (**C**) Examples of ASE events that are affected by either EBNA2, EBNA-LP or both. Gene names are indicated on the left of each panel, together with the number of the concerned exon (ex: exon). Small black arrow heads indicate the expected migration location of each isoform as determined using the FasterDB database. The last lane of each panel (/) corresponds to a PCR amplification control performed in the absence of cDNA. The relative amounts indicated under each panel represent the percentage of inclusive isoform and were quantified using Image Lab software (Bio-Rad). The highlighted panel signals a splicing event that will be further studied as a putative target for EBNA2 and EBNA-LP regulation.

First, the levels of EBNA2 and EBNA-LP mRNAs were compared between cells infected with wild-type EBV and cells infected with EBNA2- or EBNA-LP-deficient mutant EBVs. The EBNA2-KO EBV carries a point mutation in the translational start codon and our primers did not distinguish between wild-type and mutant EBNA2 transcripts in infected B cells but detected very similar amounts of EBNA2-specific RT-PCR products suggesting that the efficiency of infection of the three viruses was comparable (Figure 4A). The EBNA-LP KO EBV was generated by introducing translational stop codons in each copy of the EBNA-LP W1 exons (10). To amplify the EBNA-LP transcripts, we used a primer pair, one primer of which partially hybridizes in the region which has been mutated in the EBNA-LP KO, thereby allowing amplification of wild-type but not mutant EBNA-LP transcripts. As expected, no EBNA-LP mRNA was amplified in cells infected with the EBNA-LP KO EBV. However, it is to be noticed that more EBNA-LP transcripts were produced in cells infected with the EBNA2-KO virus than in cells infected with the wild-type virus, corroborating previous findings from Szymula *et al.* (9). Therefore, any differential effect that will be observed between the EBNA2-KO and wild-type virus infection, might be attributed not only to the absence of EBNA2 but potentially also to an increase in EBNA-LP.

Second, changes in the ratio of specific RNA isoforms of our panel of validated genes were compared between cells infected with wild-type EBV and cells infected with the EBNA2 or EBNA-LP KO EBVs. In most cases, infection with any of the three viruses induced similar changes in the alternative splice variant expression compared with uninfected cells, indicating that most changes did not specifically depend on EBNA2 or EBNA-LP expression. Three examples of these are shown in Figure 4B. Interestingly however, in 10 cases—ANKZF1, BOD1, HNRNPH3, IRF7, IVNS1ABP, MTA1, NAA25, NUMB, RBMXL1 and TLR1O—infection with EBNA2 or EBNA-LP KO EBVs, appeared to have a reduced effect on the isoform ratio when compared with infection with wild-type EBV (Figure 4C).

Taken together, these results suggest that at least part of the changes in the alternative splicing pattern observed upon infection of primary B-lymphocytes by EBV depends upon the expression of EBNA2 and/or EBNA-LP.

### Both EBNA2 and EBNA-LP promote inclusion of the *Numb* alternative exon 11 in a reporter minigene system

There are several non-exclusive modes of action by which EBNA2 and EBNA-LP could affect alternative splicing. First, the function of EBNA2 in transcriptional regulation has already been well documented. Although the function of EBNA-LP in regulating gene expression is less well studied there is some evidence that it can act as a co-activator of EBNA2 (reviewed in (38)). Therefore, EBNA2 and EBNA-LP together could modify the level of expression of various splicing factors or regulators that could indirectly alter the splicing pattern of specific targets. Second, it has been shown that the rate of RNA polymerase II (RNAPII) elongation as well as the epigenetic environment can affect splicing of a nascent RNA molecule (39). Thus, by directly regulating the expression of specific cellular genes at the level of transcription initiation and elongation (40) EBNA2 and EBNA-LP could affect the splicing efficiency of certain alternative exons. Third, EBNA2 and EBNA-LP could also affect splicing by directly interfering with the splicing machinery and splicing factors. In support of this latter hypothesis, two independent proteomic studies have identified several splicing regulators that are complexed with EBNA-LP (41,42) (Chelouah & Wiels; personal communication).

**Figure 5.**
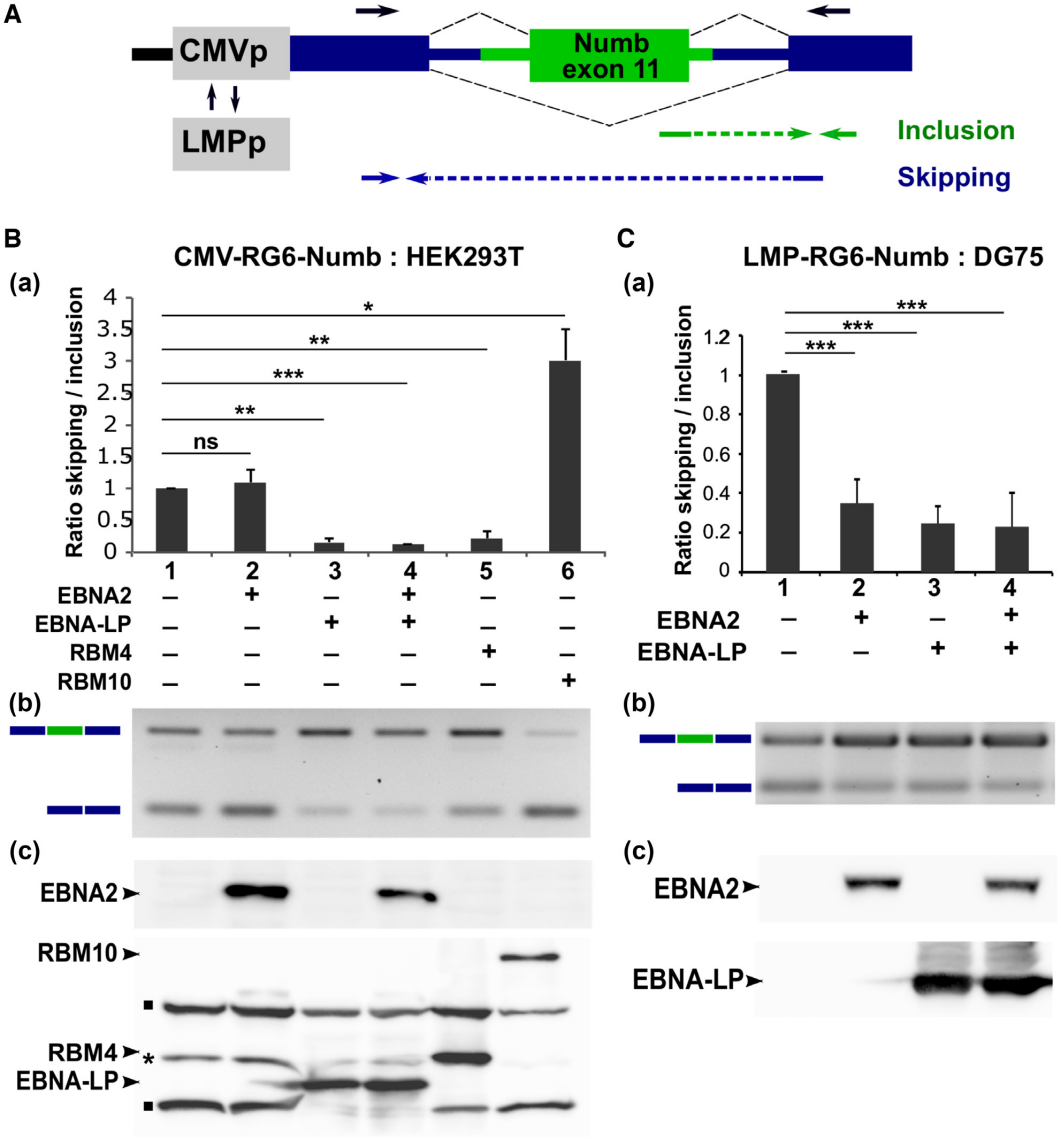
modulation of Numb exon 11 alternative splicing by EBNA2 and EBNA-LP. (**A**) Schematic representation of the RG6-Numb reporter construct (32). The green box and lines represent the human *NUMB* exon 11 and 100/50 nucleotides of the flanking 5′/3′ intronic regions, respectively. Dark blue boxes and lines indicate constitutive exons and intronic sequences specific for the RG6 minigene (34). The reporter gene is under the transcriptional control of either the cytomegalovirus immediate early promoter (CMVp) or the EBV LMP viral promoter (LMPp). Arrows placed above the RG6-Numb schematic sequence indicate the location of primers used for semi-quantitative RT-PCR. Arrows placed below indicate the location of primers used for quantitative RT-PCR (RTqPCR) to amplify specifically the inclusive or skipping isoforms. HEK293T (**B**) or DG75 B cells (**C**) were transfected with the pCMV-RG6-Numb or pLMP-RG6-Numb reporter constructs respectively, together with expression plasmids coding for EBNA2, EBNA-LP, RBM4 or RBM10 as indicated in the Figures. Panels a: relative ratio between exon inclusion and skipping are estimated using quantitative RT-PCR. Error bars represent the standard deviation from three independent experiments. The *P* values of paired Student's *t*-test are reported as *P < 0.1; **P< 0.05; ***P< 0.001; ns, not significant. Panels b: agarose gel analysis of a representative semi-quantitative RT-PCR reaction using primers depicted in A (black arrows) from one of the three experiments used for the quantitative RT-PCRs. Panels c: representative western blot analysis of one of the three experiments. Upper panels: EBNA2 is revealed using an anti-EBNA2 mAb (PE2, abcam). Lower panels: RBM4, RBM10 and EBNA-LP being all tagged with the Flag epitope were revealed using anti-Flag M2 mAb (Sigma-Aldrich). N.B.: the RG6-Numb construct carries both dsRED and EGFP coding sequences that are alternatively put in frame depending on the inclusion or skipping of Numb exon 11 (34). A Flag epitope sequence placed immediately upstream of the first exon of RG6-Numb allows the detection of these two fusion proteins by anti-Flag M2 mAb as seen on the gel (bands indicated by a black square). The higher molecular weight band corresponds to the GFP in-frame fusion generated when Numb exon 11 is included, whereas the lower molecular weight band corresponds to the dsRED in-frame fusion generated when Numb exon 11 is skipped. * Unspecific band.

In order to further investigate how EBNA2 and EBNA-LP can potentially affect splicing we selected one of the alternative splicing events that we found to be dependent on the expression of both EBNA2 and EBNA-LP: the alternative splicing of *NUMB* exon 11 (Figures 2 and 4C, highlighted panel). *NUMB* exon 11 splicing also appeared to be more strongly affected by EBV infection than by B cell activation (Figure 3). The choice of this particular splicing event was mostly determined by the existence of a well-characterized minigene reporter system developed by Bechara *et al.* (32) that allows a rapid evaluation and study of the role of the viral proteins in splicing regulation. The alternative splicing of the human *NUMB* exon 11 (formerly exon 9) has been previously reported to be regulated by RBM4 (43) and the RBM 5, 6 and 10 family of proteins (32). The minigene reporter contains the human *NUMB* exon 11 flanked by its proximal flanking intronic sequences (Figure 5A) and it has been shown to recapitulate the effect of RBM6 and RBM10 on *NUMB* exon 11 alternative splicing. We therefore made use of this reporter system to investigate whether EBNA2 or EBNA-LP could modulate the alternative splicing of *NUMB* exon 11.

First, the original pCMV-RG6-Numb construct depicted in Figure 5A was transfected into HEK293T cells together with expression plasmids encoding EBNA2, EBNA-LP or both, as well as RBM10 and RBM4 as controls. RNA was assayed both by quantitative RT-PCR (RT-qPCR) using primers specific for each isoform as indicated in Figure 5A and by semi-quantitative RT-PCR using primers localized within the surrounding constant exons. As shown in Figure 5B, RBM10 significantly favoured Numb exon 11 skipping, which is consistent with published results by Bechara *et al.* (32). By contrast, RBM4—which had been reported to modulate Numb exon 11 splicing (43) but had never been assayed in the RG6-Numb minigene system—stimulated Numb exon 11 inclusion. Interestingly, and similar to RBM4, EBNA-LP—but not EBNA2—strongly stimulated Numb exon 11 inclusion in HEK293T cells.

Since EBNA2 is a transcriptional regulator, we asked whether it may affect splicing through its ability to regulate gene transcription. To test this possibility, the CMV promoter in pCMV-RG6-Numb was replaced by the EBV LMP1 promoter whose activity depends on EBNA2 (44,45) (construct pLMP-RG6-Numb depicted in Figure 5A). As EBNA2 only activates transcription from the LMP1 promoter in B cells, pLMP-RG6-Numb was transfected into the EBV-negative DG75 B cell line together with expression plasmids encoding EBNA2, EBNA-LP or both. The results, presented in Figure 5C, confirmed the effect of EBNA-LP and demonstrated that EBNA2 could also have an effect on Numb exon 11 alternative splicing when the minigene was driven by an EBNA2-reponsive promoter. EBNA2, similar to EBNA-LP favoured Numb exon 11 inclusion. In parallel, we verified that the impact of EBNA2 on splicing correlated with promoter usage rather than with the cell line: when we used pCMV-RG6-Numb in DG75, EBNA2 had no effect on the minigene splicing as in HEK293T cells (Supplementary Figure S2).

Taken together, these results suggest that both EBNA2, in a promoter-dependent manner, and EBNA-LP, in a promoter-independent manner, regulate *NUMB* exon 11 alternative splicing.

### EBNA-LP directly interacts with RBM 4, 6 and 10 proteins

**Figure 6.**
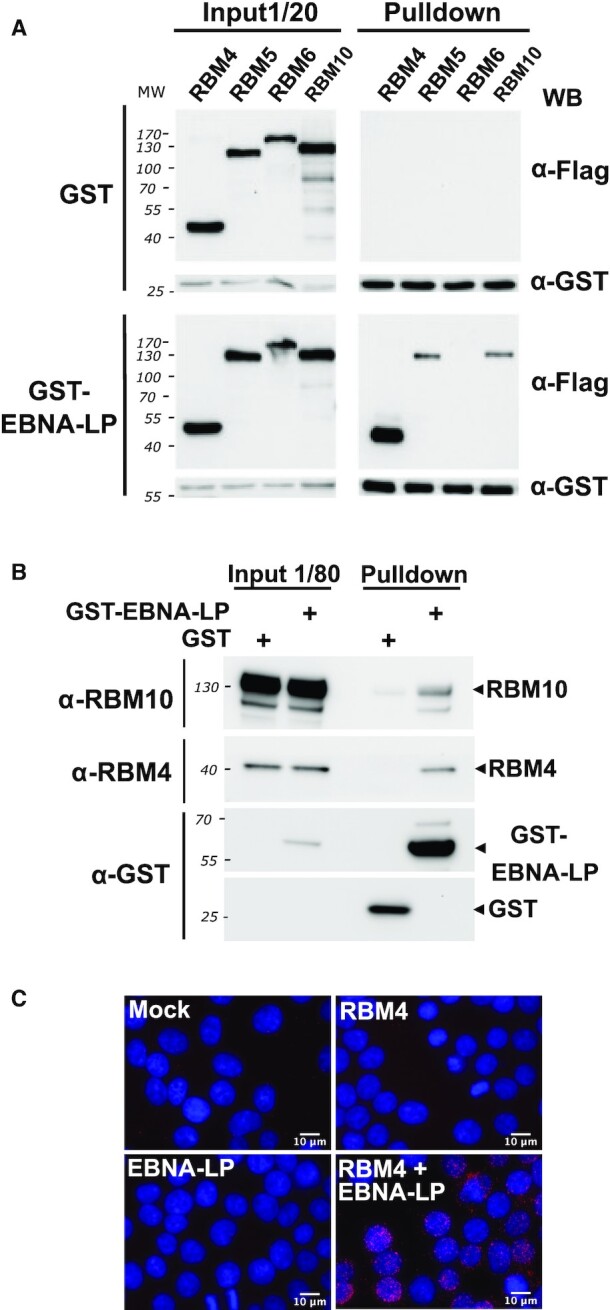
EBNA-LP interacts with both the RBM4 protein and members of the RBM5, 6 and 10 family of proteins in HEK293T cells. (**A**) RBM4 and to a lesser extent RBM5 and RBM10 proteins co-pulldown with GST-tagged EBNA-LP. Flag-tagged RBM4, RBM5, RBM6 or RBM10 expression plasmids were transfected into HEK293T cells together with either GST or GST-EBNA-LP expression plasmids. Cellular extracts were incubated with glutathione sepharose-4B beads and the pulled-down complexes analysed by western blotting using an anti-Flag antibody to detect the RBM proteins or an anti-GST antibody to detect GST and GST-EBNA-LP fusion proteins. Left panels correspond to the analysis of 1/20 of cell extract used for each pulldown prior addition of the glutathione sepharose beads. Right panels correspond to the analysis of proteins complexes pulled-down with the GST or GST-EBNA-LP proteins as indicated. (**B**) GST-tagged EBNA-LP binds endogenous RBM4 and RBM10 proteins in pulldown experiments. GST or GST-EBNA-LP expression plasmids were transfected into HEK293T as indicated. After 24 h, cellular extracts were incubated with glutathione sepharose-4B beads and the pulled-down complexes were analyzed by western blotting using anti-RBM4 or anti-GST antibodies as indicated. The two left columns correspond to the analysis of 1/80 of cell extract used for each pulldown prior to the addition of the glutathione sepharose beads. The two right columns correspond to the analysis of proteins complexes pulled down with the GST or GST-EBNA-LP proteins as indicated. (**C**) *In vivo* cell co-localization of EBNA-LP and RBM4. A Proximity Ligation Assay (PLA) experiment to determine whether EBNA-LP interacts with RBM4 intracellularly was performed with HEK293T that were mock-transfected or co-transfected with either pCI-Flag-EBNA-LP, pDEST-myc-RBM4, or both as indicated in the panels. The interaction between EBNA-LP and RBM4 was analysed 24 h post-transfection using rabbit anti-Flag and mouse anti-myc antibodies. The pictures show a maximum intensity projection of the raw image based on 20 *z*-planes. Scale bar = 10 μm. The PLA signals are shown in red together with DAPI staining in blue.

Since EBNA-LP has previously been found to interact with various splicing regulators in several independent proteomic studies (41,42) (Chelouah & Wiels, personal communication) and since *NUMB* exon 11 alternative splicing is known to be under the control of RBM4 and members of the RBM5/6/10 family of proteins, we hypothesized that EBNA-LP could act by directly interacting with these RBM proteins. To test this eventuality, we performed GST pull-down assays with protein lysates derived from HEK293T cells transiently co-transfected with expression plasmids encoding glutathione S-transferase-EBNA-LP (GST-EBNA-LP) fusion protein (or GST as a control) together with FLAG-tagged RBM4, RBM5, RBM6 or RBM10. (It should be noted that EBNA-LP in the GST-EBNA-LP construct—as well as in all EBNA-LP constructs used thereafter in this work—contains 3 W_1_–W_2_ repeats.) The pull-down assays were performed in the presence of RNAse A to ensure that protein-protein interactions were not mediated by RNA. As can be seen in Figure 6A, none of the RBM proteins were pulled down with GST alone, while RBM4, most efficiently, as well as RBM5 and RBM10 were specifically pulled down with the GST-EBNA-LP fusion protein. It should be noticed that using longer exposure of the blots, a specific pulldown of the less expressed RBM6 protein could also be observed (Supplementary Figure S3).

In order to further characterize the interaction between EBNA-LP and both RBM4 and RBM10 (as a representative member of the RBM 5/6/10 family of proteins), we performed a second GST pull-down assay with protein lysates derived from HEK293T cells transiently transfected with expression plasmids encoding GST-EBNA-LP or GST. In this assay, endogenous RBM4 and RBM10 proteins were specifically pulled down with GST-EBNA-LP but not GST (Figure 6B).

Finally, in order to document the interaction of EBNA-LP and RBM4 within cells *in vivo*, Flag-tagged EBNA-LP and Myc-tagged RBM4 were transiently expressed in HEK293T cells and protein co-localization were analysed, both by confocal microscopy and Duolink^®^ proximity ligation assay (PLA). Confocal microscopy shows that both proteins have a diffuse localization pattern within the nucleus (Supplementary Figure S4). Microscopy analysis following the Duolink® proximity ligation assay (PLA) (Figure 6, panel C) showed a punctate pattern mainly in the nucleoplasm of transfected cells indicating a close proximity of the two proteins at multiple sites within this compartment, which supports the idea of a coordinated function in the cell nucleus.

### EBNA-LP modulates *BCL-X* alternative 5′ splice site selection

**Figure 7.**
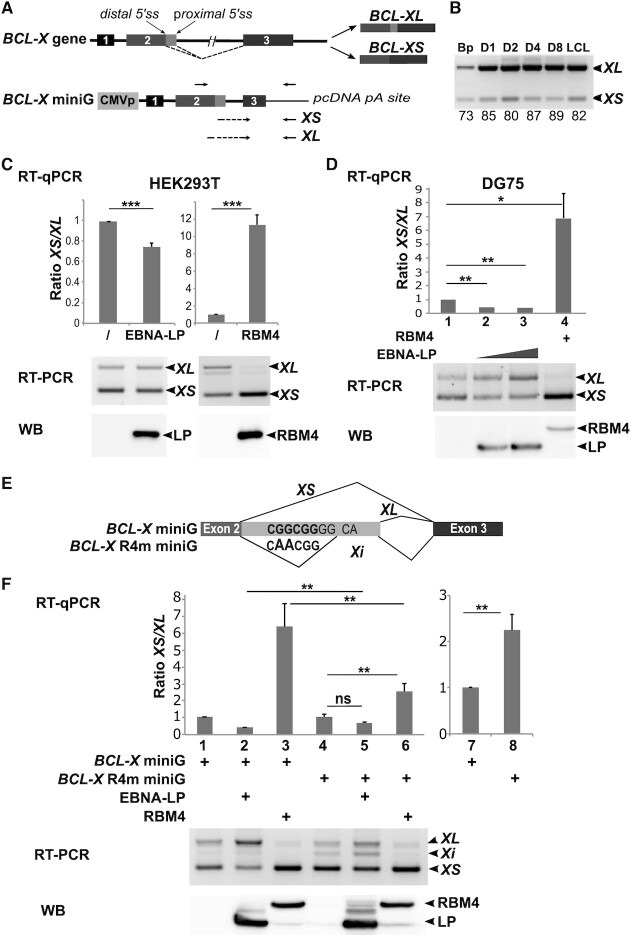
EBNA-LP modulates *BCL-X* alternative 5′ splice site selection. (**A**) Schematic representation of the *BCL-X* gene and its two alternatively spliced isoforms *BCL-XL* and *BCL-XS* and below the *BCL-X* minigene (*BCL-X* miniG) reporter construct. Exons are represented by boxes and introns by lines. Distal and proximal 5′ splice sites in exon 2 are indicated. Arrows placed above the *BCL-X* miniG schematic representation indicate the location of primers used for semi-quantitative RT-PCR. Arrows placed below indicate the location of primers used for quantitative RT-PCR to amplify either the *XL* or *XS* isoforms. (**B**) RT-PCR analysis of the respective *XL* and *XS* levels in primary B cells and EBV-infected B cells. The same RNAs as previously used to validate specific ASE events in Figure 2, served to amplify the *BCL-X* isoforms by semi-quantitative RT-PCR. PCR amplified fragments were separated on a 2.5% agarose gel. The relative amounts indicated under the panel correspond to the percentage of inclusive isoform and were quantified using Image Lab software (Bio-Rad). (**C**) EBNA-LP favors selection of the proximal 5′ splice site of the *BCL-X* miniG RNA in epithelial cells. HEK293T cells were transfected with the *BCL-X* miniG construct alone or together with an EBNA-LP or an RBM4 expression plasmid. Upper panel: the ratio between the *XS* and *XL* isoforms was analysed by quantitative RT-PCR; the data are presented as two different panels in order to better visualize the impact of EBNA-LP and RBM4 respectively on the *XS/XL* ratio compared to that obtained with the *BCL-X* miniG construct alone. Middle panel: semi-quantitative RT-PCR analysis of *XS*/*XL* levels of one representative experiment. Lower panel: western blot analysis of protein levels of one representative experiment. (**D**) EBNA-LP favours selection of the proximal 5′ splice site of the *BCL-X* miniG RNA in B cells. DG75 cells were transfected with the *BCL-X* miniG reporter construct, together with RBM4 and EBNA-LP expression plasmids as indicated. Upper, middle and lower panels are as described in C. (**E**) Schematic representation of the *BCL-X* R4m miniG mutant construct and the various RNA isoforms produced. The RBM4 binding site nucleotide sequence is indicated in bold letters. Below, the mutated RBM4 binding site nucleotide sequence is indicated with the 2 nucleotides that has been changed in larger letters. *XS*, *XL* and *Xi* RNA isoforms are represented with the thin lines indicating spliced area. (**F**) Mutation of an RBM4 binding site motif in the *BCL-X* miniG compromises the capacity of both EBNA-LP and RBM4 to regulate alternative splicing of *BCL-X* RNA. DG75 cells were transfected with mutant *BCL-X* R4m or wild-type *BCL-X* miniG constructs, together with RBM4 and EBNA-LP expression plasmids as indicated. Upper, middle and lower panels are as described in C. Left histogram (lanes 1–6): the data were normalized for each reporter. Right histogram (lanes 7 and 8) : has been generated with the same data as those used for lanes 1 and 4 of left histogram but without normalization. Error bars represent the standard deviations from a minimum of three independent experiments. The *P-*values of paired Student's t-test are indicated by the asterisks above the graphs (***P< 0.001; **P< 0.05; * P < 0.1; ns, not significant).

With the twin goals of confirming a direct role for EBNA-LP in alternative splicing regulation and further characterizing a functional relationship with RBM—the strongest interacting partner of EBNA-LP in our pulldown assays—we selected the *BCL-X* gene as a previously known target of RBM4. The product of the *BCL-X* gene (also termed *BCL2L1* or *BCL2 like 1*) is a member of the BCL-2 family of proteins that plays essential roles in apoptosis regulation. Two alternative 5′ splice sites exist in exon 2 of *BCL-X* mRNA (Figure 7A). Their alternative usage results in the translation of two protein isoforms with antagonistic effects on cell survival: the long isoform (BCL-XL) has anti-apoptotic whereas the short isoform (BCL-XS) has pro-apoptotic functions (22,46). Alternative 5′ splicing within exon 2 of *BCL-X* is regulated by several splicing regulators including RBM4 (47).

We first tested whether the regulation of *BCL-X* alternative splicing is physiologically relevant during the course of B cell infection by EBV. We confirmed that the expression of *BCL-X* is strongly enhanced in the time course of infection (4) and found that the ratio *XL* versus *XS* is increased compared to non-infected primary human B cells (Figure 7B). We next made use of a *BCL-X* reporter minigene (*BCL-X* miniG) construct (36) to investigate the impact of both EBNA-LP and RBM4 on the usage of the two alternative 5′ splice sites in exon 2 of *BCL-X*. The *BCL-X* miniG—consisting of the *BCL-X* exon 1, intron 1, exon 2, part of intron 2 and part of exon 3—is placed under the control of the CMV promoter as depicted in Figure 7A. The *BCL-X* miniG was transfected into either HEK293T (Figure 7C) or DG75 B cells (Figure 7D), together with expression plasmids encoding EBNA-LP or RBM4. In both cell types, we found that EBNA-LP consistently favoured the use of the proximal 5′ alternative splice site - leading to an increase in the *XL* isoform and a simultaneous decrease in the *XS* isoform—whereas RBM4 expression led to the opposite, a preferential use of the distal 5′ alternative splice site.

Since a binding site for RBM4 had previously been identified between the two alternative 5′ splice sites in exon 2 (47), we mutated this site as indicated in Figure 7E to yield the *BCL-X* R4m miniG construct. The mutated or unaltered *BCL-X* miniG constructs were transfected into DG75 cells alone or together with expression plasmids encoding EBNA-LP or RBM4. The semi-quantitative RT-PCR analysis showed that the mutation led to the apparition of a novel, intermediate RNA isoform that we called *Xi* (Figure 7F, RT-PCR panel, compare lanes 1 and 4). Sequencing of the *Xi* isoform revealed the use of a novel 3′ splice site located 5 nucleotides downstream of the mutation (Figure 7E). *Xi* thus appears to be a variant of the *XL* isoform that has undergone an additional splice. Hence, similar to *XL*, this variant is generated by the use of the proximal 5′ splice site and will be amplified together with *XL* in the RTqPCR experiments.

When the effect of EBNA-LP and RBM4 on the *BCL-X* and *Bcl-X* R4m miniG-derived transcripts was analyzed by RT-qPCR, we first observed, by a direct comparison of the two reporter gene constructs, that the mutation of the RBM4 binding site favoured the use of the distal alternative 5′ splice site (Figure 7F, right panel, lanes 7 and 8). This observation led us to normalize the data for each reporter gene construct separately so as to be able to compare the effect of EBNALP and RBM4 on the respective reporters directly (Figure 7F, lanes 1 and 4). Using the *Bcl-X* R4m miniG construct, the effect of RBM4 on promoting the use of the distal 5′ alternative splice site appeared to be significantly impaired, as expected, although not completely abolished (Figure 7F, compare lanes 3 and 6). Similarly, the effect of EBNA-LP on promoting the use of the proximal 5′ splice site (leading to the expression of both *XL* and *Xi* isoforms) was partially but significantly lost (Figure 7F, compare lanes 2 and 5).

Taken together, our results show that EBNA-LP can regulate the alternative splicing of *BCL-X* transcripts favouring the use of the proximal 5′ alternative splice site in exon 2 to yield the longer isoform that acts as an apoptotic inhibitor. Moreover, mutagenesis of the previously identified RBM4 binding site within exon 2 of the *BCL-X* transcript compromises the capacity of both EBNA-LP and RBM4 to regulate alternative splicing of *BCL-X* RNA.

### EBNA-LP expression induces a decrease in RBM4 protein levels

**Figure 8.**
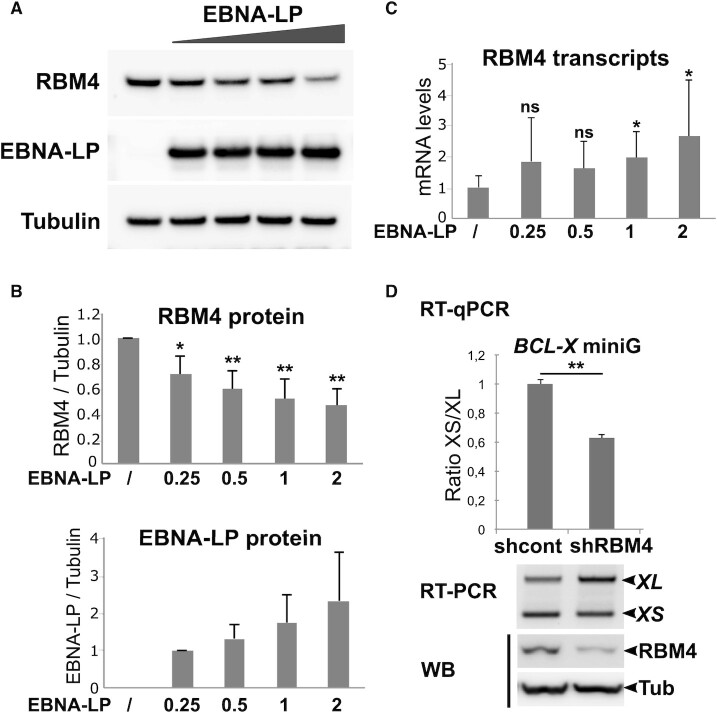
EBNA-LP expression induces a dose-dependent decrease in endogenous RBM4 protein. (**A**) HEK293T cells were transfected with increasing amounts (0.25, 0.5, 1 or 2 μg) of EBNA-LP expression plasmid for 48 h and whole cell lysates were analysed by western blot using specific antibodies against RBM4, EBNA-LP or tubulin as indicated. Using JetPEI (Polyplus transfection) as a transfecting reagent, we reach 70 to 80% of transfected cells as deduced from control transfection with a GFP expression plasmid. (**B**) Western blots obtained as indicated in panel A were quantified using Image Lab software (BioRad) with tubulin as an internal reference. Mean values from 5 independent experiments (*n* = 5) are shown with standard deviation. Significant *P-*values are indicated by asterisks above the graphs (***P< 0.001; **P< 0.05; * P < 0.1; ns, not significant). (**C**) RNA from the same cell lysates were analysed by RT-qPCR using primers specific for the RBM4 transcripts. Reported values were normalised with respects to GAPDH. (**D**) A knockdown of RBM4 using a specific shRNA leads to an increase in the use of the proximal 5′ splice site of the *BCL-X* miniG RNA. HEK293T cells were transfected with an RBM4 shRNA expression plasmid or a control shRNA plasmid for 24 h. The cells were subsequently transfected with the *BCL-X* miniG construct together with the RBM4 or control shRNA plasmids respectively for 48 h. Upper panel: the ratio between the *XS* and *XL* isoforms was analysed by quantitative RT-qPCR. Mean values of 3 replicates are shown with standard deviation. *P-*value is indicated by the asterisks above the graph (**P< 0.05); Middle panel: gel agarose analysis of a semi-quantitative RT-PCR of a representative single experiment. Lower panel: western blot analysis of tubulin and RBM4 protein levels in a representative experiment.

We next sought to understand the mechanism by which EBNA-LP can modulate *BCL-X* alternative 5′ splice site selection. Since EBNA-LP interacts with RBM4 and appears to exert an opposite effect on *BCL-X* alternative 5′ splice site selection, we first asked whether EBNA-LP expression could affect the level of endogenous RBM4 protein. To test this hypothesis, we expressed increasing amounts of an EBNA-LP expression plasmid in HEK293T cells and analysed the level of RBM4 protein by western blotting. As can be seen in Figure 8A and B, a significant and dose-dependent decrease in endogenous RBM4 protein could be observed. In parallel, the amount of RBM4 transcripts from the transfected cells did not decrease (instead, a moderate increase was seen in some experiments) after EBNA-LP expression (Figure 8C). These results suggest that EBNA-LP’s impact on *BCL-X* alternative 5′ splice site selection could at least partly be due to its capacity to lower the level of RBM4 protein in the cells.

To confirm this hypothesis, we knocked down RBM4 expression in HEK293T cells, using a specific RBM4 shRNA, before transfecting the cells with the *BCL-X* miniG construct. The changes in splicing of the *BCL-X* miniG were quantified both by RTqPCR and semi-quantitative RT-PCR followed by agarose gel analysis of the amplification products. The data presented in Figure 8D show that—similar to EBNA-LP—RBM4 shRNA knockdown leads to an increase in the amount of the *XL* isoform concomitant with a decrease in the amount of the *XS* isoform.

Taken together these results support the working hypothesis that *BCL-X* miniG splicing is regulated by RBM4 which EBNA-LP affects as it directly or indirectly reduces the steady-state level of RBM4 protein

## DISCUSSION

Several studies have characterized modifications of the cell host transcription program following EBV infection of primary B cells (4,12,13). Moreover, certain viral proteins (i.e. EBNA1, EBNA2 and the EBNA3s) that are expressed in EBV-immortalized lymphoblastoid cell lines have been characterized as transcription factors that substantially modify host cell transcription (48–54). So far, little is known about the regulation of alternative splicing of host cell transcripts following infection of primary B cells by EBV. In this study, we have characterized and identified genome-wide modifications of alternative splicing associated with EBV infection and subsequent growth transformation of infected B cells. In the course of these experiments we have also revealed a previously unknown role for the EBV EBNA-LP protein in regulating alternative splicing.

Analysis of our RNA-seq data revealed very rapid and extensive changes in alternative splice variant expression upon primary B cell infection with EBV, concomitant with dramatic transcriptional changes observed in the same time course experiments (4). A majority of the differential alternative splicing events are probably the direct consequence of the dynamic transcription program induced by EBV. First, variation in transcription efficiency can result in differential regulation of exon inclusion due to the tight coupling between transcription and splicing (55,56); second, many genes that are differentially regulated upon EBV infection encode splicing regulators (4) and could therefore strongly impact on alternative RNA splice variant expression. Third, some of the changes in splicing regulation could also be potentially due to the limited supply of specific splicing regulators in the context of an increase in transcript abundance during early phase EBV infection. However, several EBV proteins that are expressed very early upon EBV infection could also play a role. They include proteins of the replicative cycle that are expressed from viral mRNAs, that are transferred as cargo and are contained in viral particles (5) or which are transcribed from the epigenetically naïve viral genomic DNA during the so-called pre-latent phase that takes place before the viral DNA genome becomes methylated and chromatinized (57). Among the early viral genes expressed during the pre-latent phase, the product of the BMLF1 gene, a well-characterized post-transcriptional regulator with functions in mRNA export, stabilization and splicing (58,59) could participate in the regulation of cell host alternative splicing. Among the viral genes expressed during the latent phase of the cell cycle, the non-coding viral RNAs (EBER1 and EBER2) which accumulate in the nucleus in the latent phase of infection, have previously been found to alter cellular alternative splicing (60) and EBER1 has been shown to interact with the AUFI/hnRNP D splicing factor (61). EBNA1, which plays essential roles both in viral episome maintenance and viral and cellular transcriptional regulation (reviewed in (62)) has recently been found to also modulate alternative splicing of cellular genes in epithelial cells, possibly by interacting with various splicing factors, including hnRNPH1 (63,64). In addition, EBNA1 has been found in RIP-seq experiments to interact with specific cellular RNAs (64). EBNA1, which is already expressed at high levels at day 1 p.i. (4) could thus participate in extensive modification of alternative splicing during the first days of infection.

By contrast, EBNA2 and EBNA-LP have never been shown to be involved in the regulation of splicing. Using EBNA2- or EBNA-LP-KO EBVs we have now found that both proteins contribute to alternative splicing of certain specific cellular genes. Intriguingly, in the panel of genes studied, the absence of either EBNA2 or EBNA-LP led to a pattern of alternative splice variant expression which in most cases appears to be intermediate between those observed in primary B cells and wild-type EBV-infected B cells. Moreover, alternative splicing events that appear to be affected by the absence of one of the proteins are also affected by the absence of the other. This suggests that EBNA2 and EBNA-LP could functionally complement each other with regard to splicing. One possible explanation for this observation is that, on the one hand, EBNA2, as a transcriptional activator—either alone or together with EBNA-LP acting as a co-activator—participates in the regulation of expression of cellular splicing factors that themselves impact the alternative splicing of the studied genes; on the other hand, EBNA-LP, by directly interacting with some of these splicing factors—like RBM4, RBM5 and RBM10—could have an impact on the same alternative splicing events.

Among the many cellular genes that are affected by differential alternative splicing initially after EBV infection, we found a clear enrichment for factors involved in RNA processing and more specifically, RNA splicing. This observation corroborates former studies showing that splicing regulator expression is largely regulated by NMD-coupled splicing through negative or positive feedback loops. Such regulations have been suggested to be essential for maintaining cell homeostasis as well as allowing a rapid response to various stimuli (65–68). The important changes in alternative splice variant expression of RNA splicing factors induced by EBV infection probably plays an important role in the rapid switch from primary quiescent B-lymphocytes to highly proliferating lymphoblastoid cells.

Another striking finding that emerged from our analysis is the identification of a large fraction of genes (36% of our validated events) with alternative RNA isoforms predicted to be targets of NMD that are present in non-infected primary B cells. These RNAs would be expected to be eliminated by NMD but, since we detect them in primary B cells they probably escape NMD—at least partially—due to possible defects in their export from the nucleus to the cytoplasm, low level of translation (NMD is translation-dependent) or deficiency in the NMD machinery. The latter hypothesis is supported by a previous study by Tinguely *et al.* (69) in mice. The authors found a low NMD efficiency in resting B cells when compared with other B cell populations, that is strongly ameliorated upon lipopolysaccharide (LPS) activation. The accumulation of NMD-susceptible RNAs could help naïve B cells to respond more rapidly and efficiently to various physiological triggers—e.g. in germinal centers—without the need for *de novo* transcription.

Besides the changes in various alternative splicing events, we also found numerous changes in intron retention (IR) upon EBV infection. IR in mammals has only been recently acknowledged to be a common means of regulating gene expression, but the mechanisms are mostly unknown (70–72). From our GO analysis, it appears that—similar to what was observed in the case of ASE events—the list of genes affected by changes in IR level at early times of infection is enriched in genes encoding RNA splicing factors (Supplementary Figure S1). This observation is consistent with previous reports that regulatory IR particularly affects spliceosome components, splicing factors and other post-transcriptional regulators (reviewed in (71)). It is interesting to note that EBNA2 interacts with BS69, a cellular protein that at first was suggested to modulate EBNA2 function as a transcriptional activator and driver of B cell proliferation (73–75). BS69 has recently been found to regulate IR depending on its binding to H3K36me3-decorated chromatin (76), thereby opening the door to a possible—at present speculative—role for EBNA2 in IR regulation.

Clearly, many of the effects of EBNA2 and EBNA-LP on alternative splicing could be indirect and solely due to the induced cellular gene transcriptional activation by these two viral factors. However, regarding EBNA-LP, its capacity to interact with specific RNA-binding proteins, strongly suggests that it could play a more direct role in regulating alternative splicing. Among potential cellular EBNA-LP partner proteins, we characterized a potent interaction with RBM4, a multifunctional protein involved in diverse cellular processes that include alternative splicing of pre-mRNA, translation, and RNA silencing. It has been found to control apoptosis, proliferation and cell migration and to be deregulated in cancer cells (47,77,78). EBNA-LP interaction with RBM4 could therefore play important roles in the course of primary B cell infection by EBV. In line with EBNA-LP’s capacity to interact with RBM4 and other RNA splicing regulators, we identified a novel role for the protein in regulating alternative splicing using two very different models. In both cases, the alternative splicing event is also under the control of the RBM4 protein. Using the *NUMB* exon 11 model (Figure 5) the expression of both proteins favours exon inclusion, whereas in the *BCL-X* model (Figure 7) the proteins have an antagonistic effect—in contrast to RBM4, EBNA-LP expression leads to the preferred usage of the proximal 5′ splice site that can give rise to the anti-apoptotic BCL-XL protein isoform.

Whether EBNA-LP and RBM4 functionally interact to modulate alternative splicing in these two models is a key question. This is difficult to answer because the two different alternative splicing events are under the control of many splicing regulators: RBM6, RBM10 (32), RBM4 (43), ASF/SF2 and PTBP1 (79) for *NUMB* exon 11 alternative splicing; PTBP1 (80), RBM4 (47), several SR and hnRNP proteins as well as other factors that cooperate or antagonize one another (reviewed in (46)) for *BCL-X* exon 2 5′ splice site selection. However, our data suggest that one mechanism by which EBNA-LP could regulate alternative splicing, is to modulate the level of the specific splicing regulators with which it interacts, as has been found here in the case of RBM4. Regarding *BCL-X* exon 2 5′ splice site selection, the EBNA-LP-induced reduction of RBM4 protein levels is likely to account for the increase in the *BCL-XL* isoform observed in the presence of EBNA-LP (Figure 7). The situation is far less clear regarding *NUMB* exon 11 alternative splicing since both EBNA-LP and RBM4 appear to have a similar effect on *NUMB* exon 11 splicing. Thus the impact of EBNA-LP on this splicing event cannot be explained by EBNA-LP’s capacity to reduce RBM4 protein levels, but *NUMB* exon 11 splicing is also regulated by RBM10. RBM10, another interacting partner for EBNA-LP, favours *NUMB* exon 11 skipping in the context of the *NUMB* exon 11 minigene, in contrast to RBM4 (Figure 5). The fact that EBNA-LP favours *NUMB* exon 11 inclusion could suggest that, in this context, it would counteract the impact of a dominant effect of RBM10 on *NUMB* exon 11 splicing. Therefore, *NUMB* exon 11 inclusion supported by EBNA-LP is likely to result from a combination of interactions of EBNA-LP with various splicing regulators with antagonistic activities.

Taken together, our results support the view that EBV directly or indirectly regulates alternative splicing which is likely to be crucial for EBV’s success in infecting primary B cells. In this work we have studied two model genes, *NUMB* and *BCL-X*, that likely affect cell survival and proliferation in the early phase of EBV infection. In effect, *NUMB* and *BCL-X* are important modulators of cell proliferation and apoptosis respectively, and their functions depend on alternative splicing events that lead to the production of isoforms with opposing effects. NUMB is an important regulator of the NOTCH pathway. Exclusion of exon 11 generates a repressor of NOTCH and is associated with cellular proliferation, whereas inclusion of exon 11 reduces the protein level of NUMB, activating NOTCH (81–82). BCL-X long and short isoforms have antagonistic effects on cell survival (46). Importantly, EBNA-LP appears to favour the pro-proliferation isoform of NUMB and the anti-apoptotic, long isoform of BCL-X, two processes that could foster EBV-induced B cell proliferation and resistance to apoptosis especially during the hyperproliferating phase early after B cell infection.

In conclusion, our work has revealed that alternative splicing is profoundly modified during the early stages of B cell infection with EBV and unravels a novel function for the EBNA-LP protein in regulating alternative splicing of specific genes that could play a critical role in both cell proliferation and the survival of EBV-infected B cells.

## DATA AVAILABILITY

Data deposition: the raw RNA-seq data were obtained by Mrozek-Gorska *et al.* (4) and were deposited in the European Bioinformatics Insitute, ArrayExpress database, https://www.ebi.ac.uk/arrayexpress/experiments/E-MTAB-7805/ (accession no. E-MTAB-7805).

## Supplementary Material

gkab787_Supplemental_Files
