# Supplementary material for: Modulation of alternative splicing during early infection of human primary B lymphocytes with Epstein-Barr virus (EBV): a novel function for the viral EBNA-LP protein
Source: Nucleic Acids Res. 2021 Sep 16;49(18):10657–76. doi: 10.1093/nar/gkab787 (PMC8501971; doi:10.1093/nar/gkab787)

Modulation of alternative splicing during early infection of human primary B lymphocytes with Epstein-Barr virus (EBV) - a novel function for the viral EBNA-LP protein

Supplementary Data

Evelyne Manet[1*], Hélène Polvèche[2], Fabrice Mure[1], Paulina Mrozek-Gorska[3], Florian Roisné-Hamelin[1], Wolfgang Hammerschmidt[3], Didier Auboeuf[4] and Henri Gruffat[1].

1 - CIRI, International Center for Infectiology Research, Oncogenic Herpesviruses team, INSERM U1111, CNRS UMR5308, Université de Lyon, ENS de Lyon, Lyon F-69007, France.

2 - CECS, I-Stem, Corbeil-Essonnes, F-91100, France

3 - Research Unit Gene Vectors, Helmholtz Zentrum München, German Research Center for Environmental Health and German Center for Infection Research, D-81377 Munich, Germany.

4 - CNRS UMR 5239, ENS de Lyon, Lyon F-69007, France.

* To whom correspondence should be addressed: Tel.: +33 4 72 72 81 76; Fax: +33 4 72 72 81 37; Email: evelyne.manet@ens-lyon.fr

Supplementary Materials and Methods

Immunofluorescence and confocal microscopy imaging

HEK293T cells were grown on 0.1 mg/ml poly-D-lysine-coated coverslips and analyzed 24 h after transfection with expression plasmids coding for Flag-EBNA-LP and myc-tagged-RBM4. Cells were fixed with 4% paraformaldehyde for 15 min, quenched with 50 mM NH4Cl for 10 min, and permeabilized with PBS-0.1% Triton X-100 for 5 min. After a blocking step in PBS-3% BSA, cells were incubated overnight at 4°C with primary antibodies diluted in PBS-1% BSA at a dilution of 1:200 (mouse anti-myc 9E10, Covance, Inc.) and/or 1:500 (rabbit anti-Flag, Sigma-Aldrich). After three washes with PBS, cells were incubated for 1 h at room temperature with the corresponding secondary antibodies at a 1:1000 dilution: donkey anti-mouse Alexa Fluor 488 (Thermo Fisher Scientific) and/or donkey anti-rabbit Alexa Fluor 594 (Thermo Fisher Scientific). Cells were washed three times with PBS and coverslips were mounted on glass slides in Fluoromount-G medium containing DAPI (Southern Biotech). Images were acquired using a Zeiss LSM 800 Airyscan confocal laser scanning microscope and analyzed with FIJI/ImageJ software.

Supplementary Table and Figure legends

Supplementary Table 1. List of differential alternative splicing events observed between day 1 and day 0 post-infection of primary B cells with EBV. RNA-seq data from three biological replicates (indicated as R1, R2 and R3 respectively) for each condition were analyzed using FaRLine to identify alternatively skipped exons (ASE) (File tab: exon skipping), alternative 3' splice sites (A3SS) (File tab: acceptor), alternative 5' splice sites (A5SS) (File tab: donor), mutually exclusive exons (MXE) (File tab: mutually_exclusive) and multiple exons skipping (Multi Skip) (File tab: multi_exon_skipping). FDR: False discovery rate. The length difference column found in the donor and acceptor tabs corresponds to the number of additional (positive values) or missing (negative values) bases to an acceptor or donor site.

Supplementary Table 2. List of differential alternative splicing events observed between day 2 and day 0 post-infection of primary B cells with EBV. Cf. legend Table 1.

Supplementary Table 3. List of differential alternative splicing events observed between day 3 and day 0 post-infection of primary B cells with EBV. Cf. legend Table 1.

Supplementary Table 4. List of differential alternative splicing events observed between day 4 and day 0 post-infection of primary B cells with EBV. Cf. legend Table 1.

Supplementary Table 5. List of differential alternative splicing events observed between day 5 and day 0 post-infection of primary B cells with EBV. Cf. legend Table 1.

Supplementary Table 6. List of differential alternative splicing events observed between day 8 and day 0 post-infection of primary B cells with EBV. Cf. legend Table 1.

Supplementary Table 7. List of differential alternative splicing events observed between day 14 and day 0 post-infection of primary B cells with EBV. Cf. legend Table 1.

Supplementary Table 8. List of differential Intron Retention events observed upon B lymphocytes infection with EBV. RNA-seq data from three biological replicates for each condition were analyzed using the Multivariate Analysis of Transcript Splicing (rMATS) program. The different File tabs list differential intron retention events observed between each day post infection respectively and day 0.

Supplementary Table 9. List of oligonucleotides used in the study.

Supplementary Figure 1. Gene ontology (GO) enrichment analysis performed on human genes for which differential IR were identified upon EBV infection. -log10 of p-values, calculated using the Kolmogorov-Smirnov (KS) test, are represented with a color code as indicated on the right of the table. GO terms related to RNA processing or the immune response are indicated by blue and red dots respectively.

Supplementary Figure 2. Modulation of *NUMB* exon 11 splicing in the context of the pCMV-RG6-Numb reporter construct used in DG75 cells. DG75 B cells were transfected with the pCMV-RG6-Numb reporter construct together with expression plasmids coding for EBNA2, EBNA-LP or RBM10 as indicated in the Figure. Panel a: relative ratio between exon inclusion and skipping are evaluated using quantitative RT-PCR. Panel b: semi-quantitative RT-PCR analysis of exon skipping/inclusion levels

Supplementary Figure 3. EBNA-LP interacts with both the RBM4 protein and the RBM5, 6 and 10 family of proteins in HEK293T cells. Flag-tagged RBM4, RBM5, RBM6 or RBM10 expression plasmids were transfected into HEK293T cells together with either GST or GST-EBNA-LP expression plasmids. Cellular extracts were incubated with glutathione sepharose-4B beads in the presence of RNAse and the pulled-down complexes analysed by western blotting using an anti-Flag antibody to detect the RBM proteins or an

anti-GST antibody to detect GST and GST-EBNA-LP fusion proteins. Left panels correspond to the analysis of 1/20 of the cell extract used for each pulldown prior to the addition of the glutathione sepharose beads. Right panels correspond to the analysis of proteins complexes pulled down with the GST or GST-EBNA-LP proteins as indicated.

Supplementary Figure 4. HEK293T cells transiently expressing myc-RBM4 and Flag-EBNA-LP were analyzed by confocal microscopy after staining with mouse anti-myc (green) and rabbit anti-Flag (red) antibodies. Nuclei were counterstained with DAPI (blue). Representative images are shown. Scale bar = 10 μm.

Supplementary Figures

Supplementary Figure 1

Manet et al

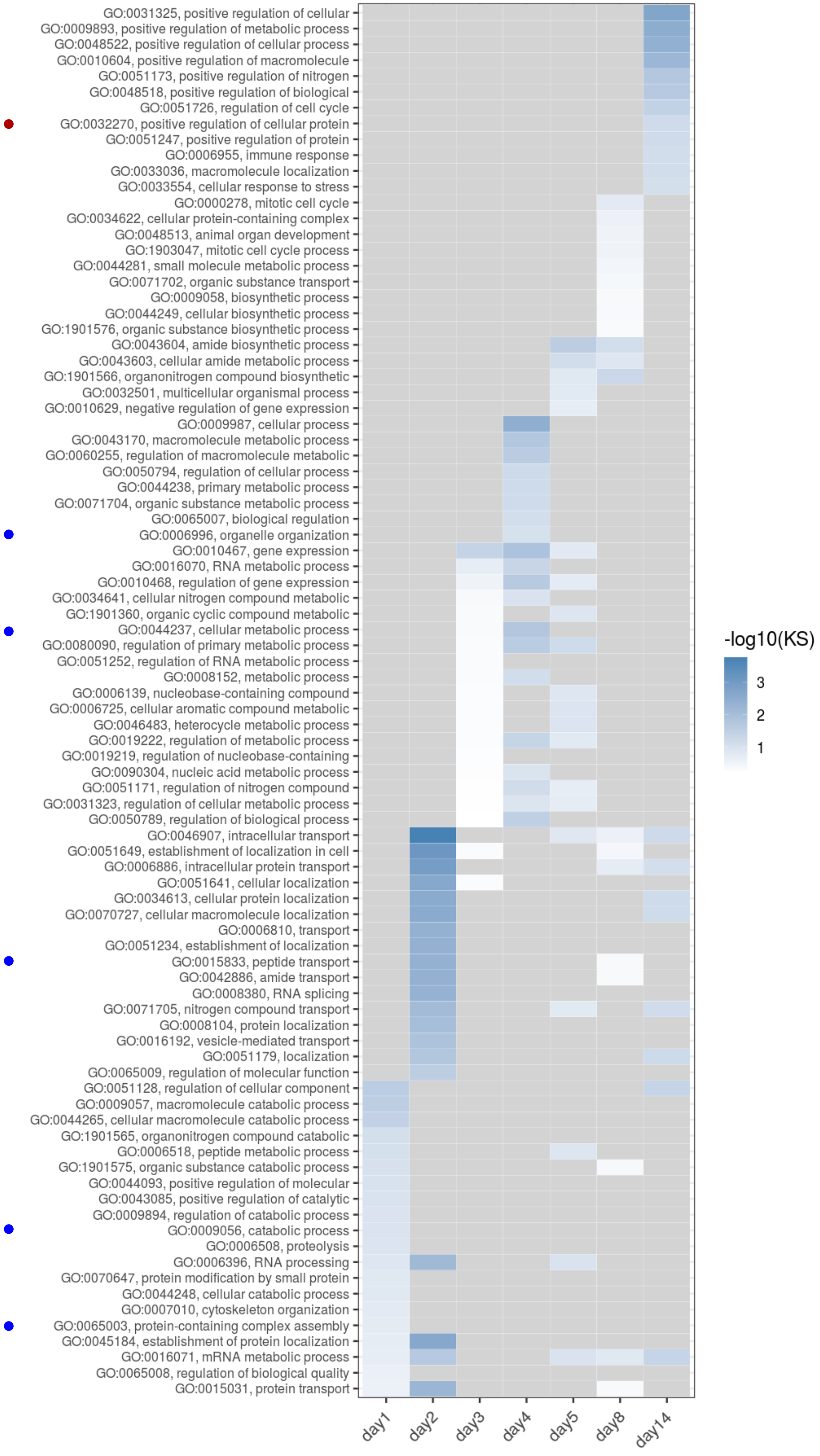

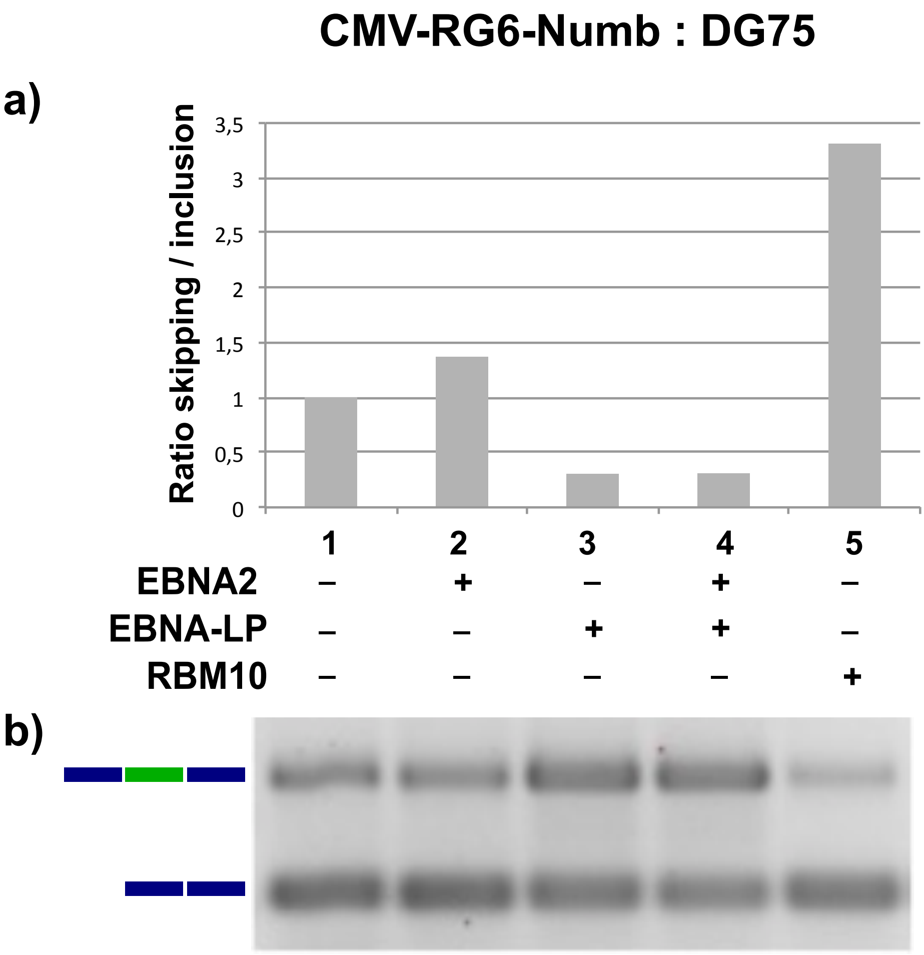
CMV-RG6-Numb : DG75
a)
Ratio skipping / inclusion
3,5
3
2,5
2
1,5
1
0,5
0
1
2
3
4
5
EBNA2 – + – + –
EBNA-LP – – + + –
RBM10 – – – – +
b)

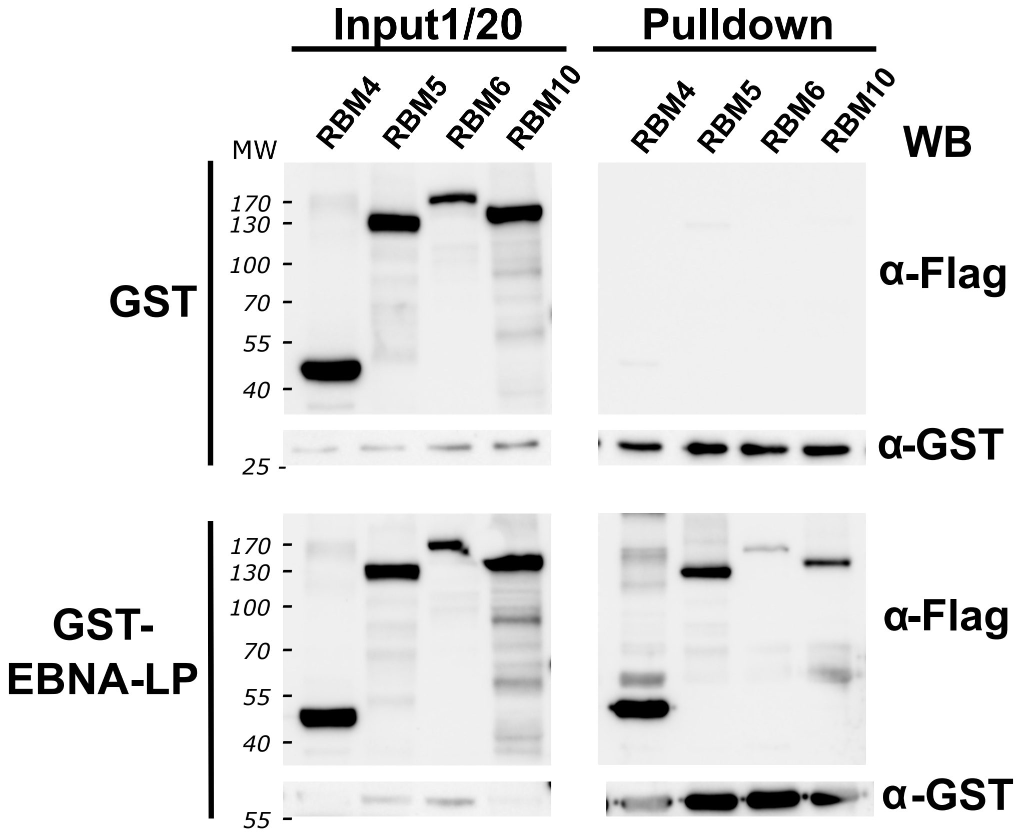
Input1/20
Pulldown
RBM4
RBM5
RBM6
RBM10
RBM4
RBM5
RBM6
RBM10
WB
MW
170
130
100
70
55
40
25
GST
α-Flag
α-GST
GST-
EBNA-LP
170
130
100
70
55
40
55
α-Flag
α-GST

Supplementary Figure 4

Manet et al

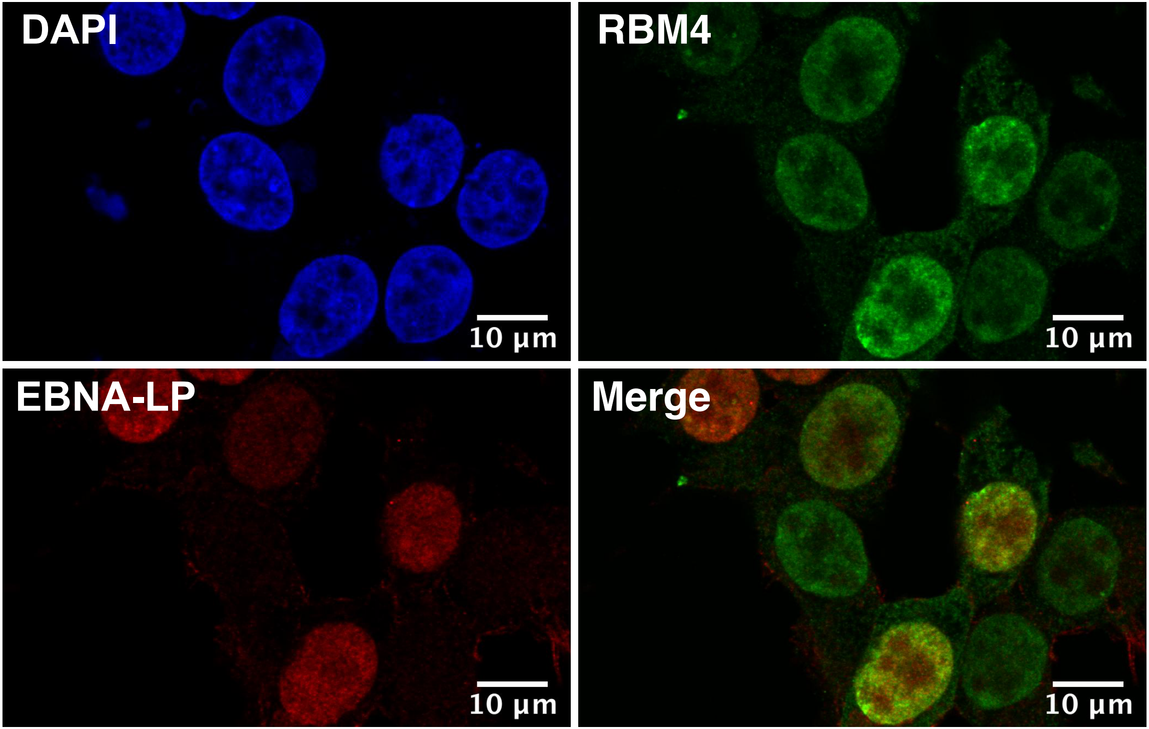

Supplement: gkab787_Supplemental_Files [file gkab787_supplemental_files.zip › Manet et al_Supplementary data.pdf]
